# Three Sample Estimates of Fraction of Missing Information From Full Information Maximum Likelihood

**DOI:** 10.3389/fpsyg.2021.667802

**Published:** 2021-08-26

**Authors:** Lihan Chen, Victoria Savalei

**Affiliations:** Psychology Department, University of British Columbia, Vancouver, BC, Canada

**Keywords:** missing data, full information maximum likelihood, regression, factor analysis, fraction of missing information

## Abstract

In missing data analysis, the reporting of missing rates is insufficient for the readers to determine the impact of missing data on the efficiency of parameter estimates. A more diagnostic measure, the fraction of missing information (FMI), shows how the standard errors of parameter estimates increase from the information loss due to ignorable missing data. FMI is well-known in the multiple imputation literature (Rubin, 1987), but it has only been more recently developed for full information maximum likelihood (Savalei and Rhemtulla, 2012). Sample FMI estimates using this approach have since then been made accessible as part of the *lavaan* package (Rosseel, 2012) in the *R* statistical programming language. However, the properties of FMI estimates at finite sample sizes have not been the subject of comprehensive investigation. In this paper, we present a simulation study on the properties of three sample FMI estimates from FIML in two common models in psychology, regression and two-factor analysis. We summarize the performance of these FMI estimates and make recommendations on their application.

## 1. Introduction

Missing data can often occur in psychological research, whether due to dropouts in longitudinal studies, skipped questions in surveys, or equipment limitations (e.g., eye-trackers failing to capture certain eye movements). Missing data can also result from the strategic use of a planned missing data design (Graham et al., 2006). For example, in studies with prohibitively lengthy questionnaires, researchers may want to administer only a subset of the questions to each participant, and treat the rest as missing, in order to reduce survey fatigue. Traditionally, psychological researchers who encounter missing data would most typically apply *ad-hoc* missing data techniques, such as *listwise deletion* or *pairwise deletion*. These methods are advantageous in their expedience, but will often lead to inconsistent estimates and power loss, resulting in bad inference. In the past decade or so, modern missing data techniques with better statistical properties, such as *full information maximum likelihood* (FIML; Allison, 1987; Muthén et al., 1987; Arbuckle, 1996) and *multiple imputation* (MI; Rubin, 1987), have become increasingly accessible via computer programs like *lavaan* (Rosseel, 2012) and *mice* (van Buuren and Groothuis-Oudshoorn, 2011), available in R (R Core Team, 2019).

Rubin (1976) defined three types of missing data mechanisms: *missing completely at random* (MCAR), when the reason data are missing is independent of any variable in the dataset, whether missing or observed; *missing at random* (MAR), when the reason data are missing is dependent only on observed data known as *conditioning variables*; and *missing not at random* (MNAR), when the reason data are missing is dependent on data that are not observed, even after conditioning on observed data. When data are missing in psychological studies, researchers typically assume ignorable missing data, which refers to MCAR or MAR missing mechanism, plus an additional technical assumption that the parameters describing the missing mechanism are independent of the model parameters (Little and Rubin, 2002). This article will focus on the impact of ignorable MAR mechanism on FIML parameter estimates. When describing the possible impact of missing data, researchers typically report only the rate of missing data per variable, in the dataset as a whole, or the proportion of incomplete cases. However, the missing rates alone are insufficient to determine the efficiency of the estimates under a general MAR mechanism.

The MAR assumption is very general, and there is a wide range of specific missing mechanisms that can generate MAR data. For example, the relationship between the conditioning variable and the missing probability can be monotonic or not monotonic (linear vs nonlinear MAR; see Collins et al., 2001; Yoo, 2009; Savalei and Rhemtulla, 2017). Further, the missing mechanism can be more deterministic or more probabilistic (strong vs weak MAR; see Yucel et al., 2011; Sullivan et al., 2018; Chen et al., 2020). When MAR mechanisms differ, the statistical properties of the FIML parameter estimates can differ even when the missing rates are the same. Although MCAR, a special case of MAR, does not involve any conditioning variables, thus avoiding some of these issues, it could still lead to parameter estimates with varying efficiency when the number of missing data patterns differ (e.g., Savalei and Bentler, 2005; Savalei and Falk, 2014).

Differential performance of FIML estimates under different MAR mechanisms can translate into differences in efficiency, and therefore different power when used to perform null hypothesis tests. In light of the replication crisis, there has been a renewed call for researchers to better understand the power of their analyses. The impact of missing data on power can only be understood if we adopt a more informative diagnostic measure than missingness rates. In this article, we argue that the appropriate diagnostic measure is a quantity known as the *fraction of missing information* (FMI). We evaluate the performance of several estimates of FMI in the context of FIML estimation within the structural equation modeling (SEM) framework. This framework is quite general and includes regression models, path analysis, confirmatory factor analysis, and general linear models involving any combination of observed and latent variables.

SEMs are usually fit as *mean and covariance structure models*. Given a set of *p* variables, a particular model implies that the mean and covariance structure of these variables is given by μ(θ) and Σ(θ), where θ is a *q* × 1 vector of model parameters. Under the assumptions that the missing data mechanism is MAR and that data are multivariate normally distributed, the FIML parameter estimates ${\hat{\theta }}_{\text{FIML}}$ can be obtained by maximizing the observed data normal-theory log-likelihood log*L*(θ|*Y*), given by (Little and Rubin, 1987):

(1) $$\begin{array}{r} \text{log}L\left(\theta |Y\right)=\overset{N}{\underset{i=1}{\sum }}\text{log}L_{i}\left(\theta |Y\right)\\ \;=-\overset{N}{\underset{i=1}{\sum }}\frac{p_{i}}{2}\text{log}\left(2\pi \right)-\frac{1}{2}\overset{N}{\underset{i=1}{\sum }}\text{log}|\Sigma_{i}\left(\theta \right)|\\ \;-\frac{1}{2}\overset{N}{\underset{i=1}{\sum }}{\left(x_{i}-\mu_{i}\left(\theta \right)\right)}^{\prime }\Sigma_{i}^{-1}\left(\theta \right)\left(x_{i}-\mu_{i}\left(\theta \right)\right), \end{array}$$

where *N* is the sample size, *p*_*i*_ is the number of variables observed for case *i*. μ_*i*_(θ) is the *p*_*i*_ × 1 model-implied vector of means, and Σ_*i*_(θ) is the *p*_*i*_ × *p*_*i*_ model-implied covariance matrix, both for the variables that are not missing in case *i*.

Parameter estimates ${\hat{\theta }}_{\text{FIML}}$ are asymptotically fully efficient if the distributional assumptions are met. However, they are not as efficient as they would have been had there been no missing data. If for the same sample of *N* observations, we write *X* = (*Y, Z*), where *Y* is the observed data, *Z* is the missing data, and *X* is the complete data, then the hypothetical complete data parameter estimates ${\hat{\theta }}_{\text{ML}}$ obtained by maximizing *L*(θ|*X*) would be more efficient than the FIML parameter estimates ${\hat{\theta }}_{\text{FIML}}$ to the extent that the missing data *Z* contains information about θ.

### 1.1. Fraction of Missing Information

The FMI measure quantifies the amount of information missing in the estimation of a particular parameter (i.e., element of θ) by considering the drop in efficiency when that parameter is estimated from the observed data *Y* rather than from the hypothetical complete data *X*. Although the FMI computation was originally introduced in the context of MI (Rubin, 1987), its theoretical roots can be traced back to the *missing information principle*, laid out by Orchard and Woodbury (1972). This principle states that the likelihood of the complete data, *L*(θ|*X*), can be factored into the likelihood of the observed data, *L*(θ|*Y*), and the density of the missing data given the observed data, *f*(*Z*|θ, *Y*):

(2) $$\begin{array}{r} L\left(\theta |X\right)=L\left(\theta |Y\right)f\left(Z|\theta ,Y\right). \end{array}$$

It follows that the first derivative of the complete data log-likelihood, or the *score* vector, can be written as

(3) $$\begin{array}{r} \frac{\partial \text{log}L\left(\theta |X\right)}{\partial \theta^{\prime }}=\frac{\partial \text{log}L\left(\theta |Y\right)}{\partial \theta^{\prime }}+\frac{\partial \text{log}f\left(Z|\theta ,Y\right)}{\partial \theta^{\prime }}. \end{array}$$

The information matrix can be defined as the covariance matrix of the score vector (Rao, 1973). Let $J_{X}=Cov\left(\frac{\partial \text{log}L\left(\theta |X\right)}{\partial \theta^{\prime }}\right)$, $J_{Y}=Cov\left(\frac{\partial \text{log}L\left(\theta |Y\right)}{\partial \theta^{\prime }}\right)$, and $J_{X|Y}=Cov\left(\frac{\partial \text{log}f_{2}\left(Z|\theta ,Y\right)}{\partial \theta^{\prime }}\right)$, the missing information principle can then be stated as

(4) $$\begin{array}{r} J_{X}=J_{Y}+J_{X|Y}. \end{array}$$

When the model is correctly specified and the multivariate normality assumption is met, it is the case that $J_{X}=-𝔼\frac{\partial^{2}\text{log}L\left(X|\theta \right)}{\partial \theta \partial \theta^{\prime }}$, i.e., the information matrix for complete data is also the negative expectation of the second derivative of the log-likelihood of the data (Rao, 1973). Similarly, $J_{Y}=-𝔼\frac{\partial^{2}\text{log}L\left(Y|\theta \right)}{\partial \theta \partial \theta^{\prime }}$ is the information matrix for observed data. The quantity *J*_*X*|*Y*_ is known as *missing information*. Equation (4) states that complete information can be written as a sum of observed information and missing information.

When the distributional assumptions are met and the model is correct, the asymptotic covariance matrix of the FIML estimates ${\hat{\theta }}_{\text{FIML}}$ (multiplied by $\sqrt{n}$ to stabilize the asymptotic distribution^1^) is given by $J_{Y}^{-1}$, whereas the asymptotic covariance matrix of the hypothetical ML estimates ${\hat{\theta }}_{\text{ML}}$ from complete data is given by $J_{X}^{-1}$. The increase in the sampling variability of the *j*th parameter estimate (i.e., the *j*th element of ${\hat{\theta }}_{\text{FIML}}$) due to missing data is therefore ${\left\{J_{Y}^{-1}\right\}}_{jj}-{\left\{J_{X}^{-1}\right\}}_{jj}$, where the subscript *jj* indicates *j*th diagonal element of a matrix. The FMI for the *j*th parameter estimate ${\hat{\theta }}_{\text{FIML},j}$, can then be defined as the proportion of its sampling variability that is due to missing data (Savalei and Rhemtulla, 2012):

(5) $$\delta_{j}=\frac{{\left\{J_{Y}^{-1}\right\}}_{jj}-{\left\{J_{X}^{-1}\right\}}_{jj}}{{\left\{J_{Y}^{-1}\right\}}_{jj}}=1-\frac{{\left\{J_{X}^{-1}\right\}}_{jj}}{{\left\{J_{Y}^{-1}\right\}}_{jj}}=1-\frac{S{𝔼}_{j,C}^{2}}{S{𝔼}_{j,O}^{2}},$$

where *SE*_*j, O*_ is the standard error of ${\hat{\theta }}_{\text{FIML},j}$, and *SE*_*j, C*_ is what the standard error would have been under complete data (i.e., the standard error of the hypothetical estimate ${\hat{\theta }}_{\text{ML},j}$ that would have been obtained from complete data).

Savalei and Rhemtulla (2012) gave an applied interpretation of the FMI in terms of its relationship to the *width inflation factor* (WIF). The WIF is the increase in the standard error and the width of the confidence interval due to missing information. Define the WIF for the *j*th parameter as ${\text{WIF}}_{j}=\frac{SE_{j},O}{SE_{j},C}$. Then, from Equation (5), we have the relationship ${\text{WIF}}_{j}=\frac{1}{\sqrt{1-\delta_{j}}}$. This equation shows that there is a one-to-one correspondence between the FMI and the impact missing data have on the property of the parameter estimate. For example, when δ_*j*_ =.75, WIF_*j*_ = 2, which means the standard error of the *j*th parameter is twice as large as it would have been under complete data. Consequently, the confidence interval of that parameter estimate also becomes twice as wide. When δ_*j*_ =.5, the same computation gives us WIF_*j*_ = 1.5, which shows the standard error and the CI are 50% larger due to missing data.

### 1.2. Sample Estimates of FMI

To obtain sample estimates of FMIs following FIML estimation from Equation (5), which provides the population definition, we need standard error estimates corresponding to complete and incomplete data. However, as we only have incomplete data, it may appear that we only have standard error estimates based on incomplete data. To bypass this problem, Savalei and Rhemtulla (2012) proposed to estimate what the complete data standard errors for each parameter would have been by evaluating the theoretical formulae for ML standard errors under complete data at the FIML parameter estimates. To do so, the model-implied means and covariances $\mu \left({\hat{\theta }}_{\text{FIML}}\right)$ and $\Sigma \left({\hat{\theta }}_{\text{FIML}}\right)$, are first obtained by analyzing the data via FIML. SEM softwares are then able to use these means and covariances to perform a complete data estimation routine, which produces standard errors to be used as complete data standard error estimates in the computation of FMI. This computation appeared to work well, and has since been automated in *lavaan*. However, the initial implementation had problems, in that negative estimates of FMIs were often produced, particularly in small samples. The reason for this poor performance was that by default, SEM software packages compute so-called “expected” standard errors for complete data and “observed” standard errors for incomplete data (Savalei, 2010), and the estimates proposed and studied in Savalei and Rhemtulla (2012) relied on these defaults. However, in small samples, the differences between these types of standard errors can be large enough to create problems, resulting in negative FMI estimates. Starting with *lavaan* version 0.6-7, the package now uses the same type of information matrix, leading to the same type of standard error estimates, when estimating FMIs using Equation (5)^2^. Because standard errors for incomplete MAR data are not consistent unless they are based on observed information (Little and Rubin, 2002), we recommend always using observed information to compute FMIs; this is in fact the default in *lavaan*.

There are three computational variations that fall under the broad label of “observed” standard errors, all of which remain consistent when the missing mechanism is MAR and the model is true. These three computational variations will be identical for saturated models (i.e., models that have zero degrees of freedom, such as regression models), but they will generally differ for structured models, i.e., models that have positive degrees of freedom and that impose some constraints on the means and/or the covariance matrix of the data. These variations are described in Appendix B, and they are implemented in *lavaan* version 0.6-7 (for further details, see Savalei and Rosseel, ress). These variations lead to three different computational estimates of FMI, which we will call ${\hat{\delta }}_{1,j}$, ${\hat{\delta }}_{2,j}$, and ${\hat{\delta }}_{3,j}$. Briefly, the estimate ${\hat{\delta }}_{1,j}$ uses the numeric Hessian (the second derivative of the log-likelihood) in both complete and incomplete standard error estimates. Standard errors based on the Hessian are the default in *lavaan* when the FIML estimator is requested, and these will be the default FMI estimates. The second estimate, ${\hat{\delta }}_{2,j}$, is obtained analytically by approximating the second derivative of the log-likelihood with the first (dominant) term and dropping the second term that depends on the second derivatives of the model (this second smaller term is cumbersome to obtain analytically). The third estimate, ${\hat{\delta }}_{3,j}$, has the same analytic form as ${\hat{\delta }}_{2,j}$, but uses unstructured estimates of means and covariance matrix in all parts of the computation that rely on these estimates.

When the proposed structured model is true, the three estimates are consistent estimates of the same quantity and therefore asymptotically equivalent (see Appendix B; Savalei and Rosseel, ress). However, they will diverge when the model is false, and only the estimate ${\hat{\delta }}_{1,j}$, based on the Hessian, will remain consistent. As this is the first study of these estimates, we only evaluate the scenarios where the proposed model is either saturated or true. In such situations, the estimate ${\hat{\delta }}_{1,j}$ may have the disadvantage that it is more complicated to compute and may thus lead to less accurate FMI estimates in small samples, but this is a prediction that needs to be empirically evaluated.

## 2. Simulation Study

While the idea for how to obtain sample FMIs following FIML estimation was published a number of years ago (Savalei and Rhemtulla, 2012), we are not aware of any simulation studies that have been conducted to evaluate the performance of sample FMIs. Furthermore, it was not until recently that FMI estimates with better properties (using the same type of standard errors in both numerator and denominator), have become automated in *lavaan*. In order to evaluate the performance of these sample FMI estimates, we conducted a simulation study with two commonly used models in psychology, a simple regression model and a two-factor model. The main objective was to determine whether the population FMI values can be estimated with reasonable accuracy under a realistic sample size. The three estimates, ${\hat{\delta }}_{1,j}$, ${\hat{\delta }}_{2,j}$, and ${\hat{\delta }}_{3,j}$, were expected to be practically equal in the saturated regression model. It was unclear, however, how they would differ in the two-factor model. Therefore, another objective for the study was to compare their performance.

### 2.1. Method

The study was conducted in *R* v3.6.1 (R Core Team, 2019). All FMIs were computed from *lavaan* 0.6-7 (Rosseel, 2012), using options specified in Supplementary Table 1. Example code of how to use *lavaan* to compute the three FMI estimates is given in Appendix A. Simulations were conducted with 1,000 replications in each cell. An example of the simulation code is available on OSF at https://osf.io/xyzt8/.

#### 2.1.1. Model 1: Simple Regression

The simple regression model simulations contained 4 (sample size) × 3 (missing mechanisms) × 3 (missing rates) = 12 conditions. For each condition, we examined the three sample FMIs (${\hat{\delta }}_{1,j}$, ${\hat{\delta }}_{2,j}$, ${\hat{\delta }}_{3,j}$) of the regression coefficient (β). Data were simulated from the path model *Y* = β*X*+*E*, where *X* and *Y* followed standard normal distributions (i.e., means of 0 and standard deviations of 1), β was always 0.4. *E* was therefore a normal distribution with a mean of 0 and standard deviation of $\sqrt{1-\beta^{2}}=.92$. The sample sizes were *N*=50, 100, 200, and 500. In order to compute the bias of the sample FMI estimates, pseudo-population values of the FMIs were computed from an additional single replication simulation with *N* = 1, 000, 000.

The missing data mechanism conditions were MCAR, linear MAR (MAR-L), and nonlinear MAR (MAR-NL), with values missing on *X* at population rates of 0.2, 0.4, and 0.6. To avoid the NMAR mechanism, missingness was only assigned to *X*, with missing rates π_*mis*_ = 20, 40, and 60%. Since missingness was only assigned to one of the two variables, the overall missing rates were therefore 10, 20, and 30%, respectively. In the special case of MCAR, each value of *X* was assigned an independent π_*mis*_ chance of missing. For MAR conditions, we specifically chose very strong selection mechanisms in the MAR conditions, as weak MAR mechanisms tend to behave similarly to each other and to MCAR. The choice of strong selection mechanisms allows us to contrast the differences between the different MAR conditions. For these MAR conditions, the missingness in *X* was conditioned on *Y*.

Under MAR-L, values of *X* were set to be missing with a high probability when the corresponding values of *Y* were above a certain cutoff. A single cutoff rule was used to implement this mechanism. When *Y* was above the percentile cutoff value *y*_*cut*_, the missing rate on *X* was π_1_ = 90%; below that cutoff, the missing rate on *X* was π_2_ = 10%. In each condition, the value *y*_*cut*_ was computed from the theoretical π_*c*_ percentile of the population distribution of *Y*, where π_*c*_ = (π_*mis*_−π_1_)/(π_2_−π_1_)= 0.875, 0.625, and 0.375. This produces a linear MAR mechanism with per variable missing rates of π_*mis*_ = 20, 40, and 60%, respectively. Since *Y* followed a standard normal distribution, the cutoffs were, for each missing rate condition respectively, *y*_*cut*_= 1.15, 0.32, and −0.32.

MAR-NL was simulated using two cutoffs, *y*_*cut*1_ and *y*_*cut*2_. When *Y* was either above *y*_*cut*1_ or below *y*_*cut*2_, the missing rate was π_1_ = 90%; between those two cutoffs, the missing rate was π_2_ = 10%. The cutoffs were the theoretical 50%±0.5π_*c*_ percentiles of the population distribution of *Y*, where 0.5π_*c*_= 0.4375, 0.3125, and 0.1875. Since *Y* followed a standard normal distribution, the cutoffs were, for each condition respectively, *y*_*cut*1_= 1.53, 0.89, and 0.49 and *y*_*cut*2_= −1.53, −0.89, and −0.49. This type of nonlinear MAR resulted in missing values on both ends of the distribution of *X*, which would theoretically lead to more information loss.

#### 2.1.2. Model 2

The two-factor model simulations contained 4 (sample size) × 3 (missing mechanisms) × 3 (missing rates) = 12 conditions. For each condition, we investigated the properties of the three sample FMIs (${\hat{\delta }}_{1,j}$, ${\hat{\delta }}_{2,j}$, ${\hat{\delta }}_{3,j}$) for the factor correlation (ϕ) and factor loadings (λs). The two latent factors were *F*_1_ and *F*_2_, and each factor included with four indicators: *X*_1_, *X*_2_, *X*_3_, *X*_4_ measured *F*_1_, and *Y*_1_, *Y*_2_, *Y*_3_, *Y*_4_ measured *F*_2_. All indicators, factors, and measurement errors followed standard normal distributions. The two factors were correlated at ϕ = 0.4. The measurement model was set to have equal loadings, with all loading values set to λ = 0.49 (see Supplementary Table 2 for a summary of the study design). As factor analysis is typically conducted with a larger sample size than regression, the sample sizes were set to *N*=100, 200, 500, and 1,000, excluding the *N* = 50 condition and adding the *N*=1,000 condition. The “population”-level FMIs were once again computed from a single sample with *N* = 1, 000, 000.

Three missing data mechanism conditions, MCAR, MAR-L, and MAR-NL, were included in the two-factor model simulations. Half the variables were assigned to contain missing values: *X*_2_, *X*_4_, *Y*_2_, and *Y*_4_. The variables *X*_1_, *X*_3_, *Y*_1_, and *Y*_3_ were fully observed. Where the missing data mechanism was not MCAR, missingness on *X*_2_ and *X*_4_ was conditioned on the value of *C*_*X*_ = *X*_1_+*X*_3_. Similarly, missingness on *Y*_2_ and *Y*_4_ was conditioned on the value of *C*_*Y*_ = *Y*_1_+*Y*_3_. The cutoffs for MAR-L and MAR-NL were based on the standardized values of *C*_*X*_ and *C*_*Y*_, and were the same as those in the regression model. For each variable containing missing values, the missing rate on that variable was π_*mis*_ = 20, 40, and 60%. Since only half the variables contained missingness, these per variable missing rates corresponded to overall missing rates of 10, 20, and 30%.

#### 2.1.3. Evaluation Criteria

In order to assess the properties of the sample estimates of FMIs, we computed the raw bias, root mean squared error (RMSE), and 95% equal-tailed interval (ETI) width based on the 1,000 replications. The raw bias was computed as $\bar{{\hat{\delta }}_{e,j}}-{\hat{\delta }}_{pop,j}$, where $\bar{{\hat{\delta }}_{e,j}}$ was the mean of the sample FMI estimates produced in all replications, and *e*= 1, 2, or 3, as the evaluation criteria were computed in the same when for all three FMI estimates. ${\hat{\delta }}_{pop,j}$ was the pseudo-population FMI value generated from the additional single replication with *N*= 1,000,000. The raw bias indicated how much the sample FMI deviated from the population on average, in the long run. We expected these estimates to be largely unbiased, and therefore we considered a bias of more than 0.05 (δ ranged from 0 to 1) to be notable.

The RMSE was computed as $\sqrt{\frac{1}{N_{rep}}\Sigma_{i=1}^{N_{rep}}{\left({\hat{\delta }}_{e,j,i}-{\hat{\delta }}_{pop,j}\right)}^{2}}$, where *N*_*rep*_ was the number of replications and ${\hat{\delta }}_{e,j,i}$ was the sample FMI estimate on the *i*th replication. The RMSE captured the bias and variability of the estimate from run to run, and could be roughly interpreted as the average difference from the true population value. Given that we expected the estimates to be largely unbiased, the RMSE was mainly used as a measure of efficiency.

As the RMSE is not very easily interpretable, we included an additional efficiency measure, the 95% ETI width, defined as the difference between the 2.5th quantile and the 97.5th quantile of the observed $\hat{\delta }$ values across all replications. The ETI width therefore indicated where most of the sample estimates lied across all replications, and a low ETI width suggests the parameter estimate is more precise. In the case of a symmetrical sampling distribution, 95% of the estimates in all replications fall within an interval of $\bar{{\hat{\delta }}_{e,j}}$ plus and minute half the ETI width. For example, if the mean of a symmetrical FMI sampling distribution is 0.5, an ETI width of 0.2 shows that 95% of the sample FMIs fall within, 0.5±0.2/2, or [0.4, 0.6].

### 2.2. Results

In this study, we examine the FMIs of three parameters, namely, the regression coefficient (β), the factor loading (λ), and the factor correlation (ϕ). For simplicity of notation, the numeric subscript *j* is substituted by the name of the parameter when a specific FMI is referenced. For example, the numeric Hessian FMI estimate ${\hat{\delta }}_{1,j}$ for the regression coefficient β will be denoted as ${\hat{\delta }}_{1,\beta }$. The result summary of each simulation condition is available on OSF at https://osf.io/xyzt8/.

#### 2.2.1. Population Values

As was theoretically expected, the three FMI computations were identical at *N*=1,000,000. Therefore, for the population values, we do not distinguish between δ_1, *j*_, δ_2, *j*_, and δ_3, *j*_, and simply refer to the value as δ_*pop, j*_.

In Model 1 (regression), the FMIs of the regression coefficient (β) under per variable missing rates of π_*mis*_=0.2, 0.4, 0.6, respectively, were δ_*pop, j*_=0.15, 0.33, and 0.52 under MCAR. Under MAR-L, they were δ_*pop*, β_=0.29, 0.51, 0.63, and MAR-NL, they were δ_*pop*, β_=0.35, 0.65, 0.79 (as seen in Table 1). As expected, the population FMIs increased as the missing rate increased in all three missing data mechanism conditions. However, the missing rates did not capture all the information loss. When the missing rates were equal, the FMIs could vary greatly across the three missing data mechanism conditions. Most notably, due the loss of data at the tail ends of the distribution in the nonlinear MAR condition, this missing data mechanism was associated with the highest FMIs.

**Table 1:** The population FMIs.

**Model**	**Parameter**	**π_*mis*_**	**δ_*pop, j*_**		
			**MCAR**	**MAR-L**	**MAR-NL**
Regression	β	0.2	0.15	0.29	0.35
Regression	β	0.4	0.33	0.51	0.65
Regression	β	0.6	0.52	0.63	0.79
Two-factor	ϕ	0.2	0.15	0.29	0.35
Two-factor	ϕ	0.4	0.33	0.51	0.65
Two-factor	ϕ	0.6	0.52	0.63	0.79
Two-factor	λ	0.2	0.15	0.29	0.35
Two-factor	λ	0.4	0.33	0.51	0.65
Two-factor	λ	0.6	0.52	0.63	0.79

*Numbers computed from N=1,000,000. π_mis_ is the probability of missing value in each variable with missingness. The overall missing rate is π_mis_/2. β, the regression coefficient. ϕ, the factor correlation. λ, the factor loading. The population FMIs of the four factor loadings corresponding to variables containing missingness are all within 0.01 of each other, and therefore only the FMIs of the λ for X_2_ is reported*.

In Model 2 (two-factor model), there were 4 factor loadings associated with variables that contained missing data, namely the factor loadings of *X*_2_, *X*_4_ on *F*_1_, and the factor loadings of *Y*_2_, *Y*_4_ on *F*_2_. As these loadings were of the same size, and the missing rate was the same for all four variables, only the FMI on the loading of *X*_2_ is reported here. Under MCAR, the FMIs of the factor loading (λ) were δ_*pop*, λ_=0.20, 0.40, and 0.60 for per-variable missing rates of π_*mis*_=0.2, 0.4, and 0.6, respectively. Under MAR-L, the FMIs were δ_*pop*, λ_=0.37, 0.60, 0.71. Under MAR-NL, they were δ_*pop*, λ_=0.44, 0.71, 0.83. Once again, we observed that in each condition, the FMI increased as the missing rates increased, and the MAR-NL condition lead to the highest FMIs, followed by MAR-L, and then MCAR.

The pattern of differences between the missing data mechanisms, however, did not hold for the FMIs of the factor correlation (ϕ) in Model 2. The FMIs under per variable missing rates of π_*mis*_=0.2, 0.4, and 0.6, respectively, were δ_*pop*, ϕ_=0.13, 0.26, 0.38 in MCAR, δ_*pop*, ϕ_=0.14, 0.27, 0.38 in MAR-L, and δ_*pop*, ϕ_=0.14, 0.28, and 0.39 in MAR-NL. The FMIs of the factor correlation, unlike the FMIs of other parameters, did not differ notably across the missing mechanisms. Even within the same model, parameters affected by the same missing rates and the same missing data mechanism could see drastically different amounts of information loss. For instance, under π_*mis*_ = 0.2 and MAR-NL, the FMI on ϕ was δ_*pop*, ϕ_ = 0.14, but the FMI on λ in the same model was δ_*pop*, ϕ_ = 0.44. These results emphasize the point that information loss is not predictable from the missing rates and whether the missing data mechanism is MCAR or MAR. Information loss can only be quantified by computing the FMI for a particular mechanism and parameter of interest.

#### 2.2.2. Model 1

For the regression coefficient β, the three sample FMI estimates, ${\hat{\delta }}_{1,\beta }$, ${\hat{\delta }}_{2,\beta }$, and ${\hat{\delta }}_{3,\beta }$ were identical, due to the regression model being saturated. We refer to the resulting estimate simply as ${\hat{\delta }}_{\beta }$ in Table 2. As we can see in the tables, the sample FMI estimates were largely unbiased, with the long run means of ${\hat{\delta }}_{\beta }$ over the 1,000 replications falling within 0.05 of the population value in practically all conditions. Notable bias (i.e., >0.05) only arose when the missing data mechanism was NL-MAR, the sample size was small, and the missing rate was high, where the sample FMI could underestimate the population value by as much as 0.07 (near the top of Table 2 under the MAR-NL columns).

**Table 2:** The bias, RMSE, and the 95% equal-tailed interval width of the regression coefficient FMI estimate (${\hat{\delta }}_{\beta }$).

**π_*mis*_**	**N**	**MCAR**			**MAR-L**			**MAR-NL**		
		**Bias**	**RMSE**	**ETI**	**Bias**	**RMSE**	**ETI**	**Bias**	**RMSE**	**ETI**
0.2	50	0.01	**0.07**	**0.28**	−0.01	**0.12**	**0.45**	−0.02	**0.14**	**0.52**
0.4	50	0.00	**0.10**	**0.39**	−0.02	**0.15**	**0.54**	**−0.05**	**0.19**	**0.64**
0.6	50	0.00	**0.11**	**0.43**	−0.02	**0.14**	**0.52**	**−0.07**	**0.19**	**0.57**
0.2	100	0.00	0.05	0.19	0.00	**0.09**	**0.34**	−0.01	**0.10**	**0.39**
0.4	100	0.00	**0.07**	**0.28**	−0.01	**0.11**	**0.43**	−0.03	**0.15**	**0.56**
0.6	100	0.01	**0.08**	**0.30**	−0.01	**0.11**	**0.42**	**−0.06**	**0.16**	**0.53**
0.2	200	0.00	0.03	0.13	0.00	**0.06**	**0.25**	0.00	**0.08**	**0.30**
0.4	200	0.00	0.05	0.19	−0.01	**0.09**	**0.35**	−0.02	**0.12**	**0.45**
0.6	200	0.00	**0.06**	**0.22**	0.00	**0.08**	**0.31**	−0.03	**0.12**	**0.44**
0.2	500	0.00	0.02	0.09	0.00	0.04	0.16	0.00	0.05	0.19
0.4	500	0.00	0.03	0.12	0.00	**0.06**	**0.22**	−0.01	**0.08**	**0.29**
0.6	500	0.00	0.04	0.14	0.00	**0.05**	**0.21**	−0.01	**0.07**	**0.28**

*π_mis_ is the probability of missing value in each variable with missingness. The overall missing rate is π_mis_/2. ETI denotes the 95% ETI width, which is the distance between the 2.5 and 97.5 percentile of the sampling distributions. Bias values with magnitudes >0.05, RMSEs >0.05, and ETI widths >0.20 are indicated with bold fonts. The conditional formatting was applied prior to rounding*.

The sample FMI estimates were not particularly efficient at the smaller sample sizes typical of regression analyses, and would produce highly variable results from run to run. At *N* = 50 and 100, the RMSEs were around 0.10 or higher across nearly all missing rates and missing data mechanisms. At *N* = 100, the estimate was the most precise estimate under MCAR with π_*mis*_ = 0.2, with RMSE of 0.05 and 95% ETI width of 0.19. For larger sample sizes *N* = 200 and 500, the performance of ${\hat{\delta }}_{\beta }$ was overall acceptable under MCAR, but not for MAR-L and MAR-NL. Under both MAR conditions, good performance was only achieved when *N* = 500 and π_*mis*_ = 0.2.

**Figure 1:**
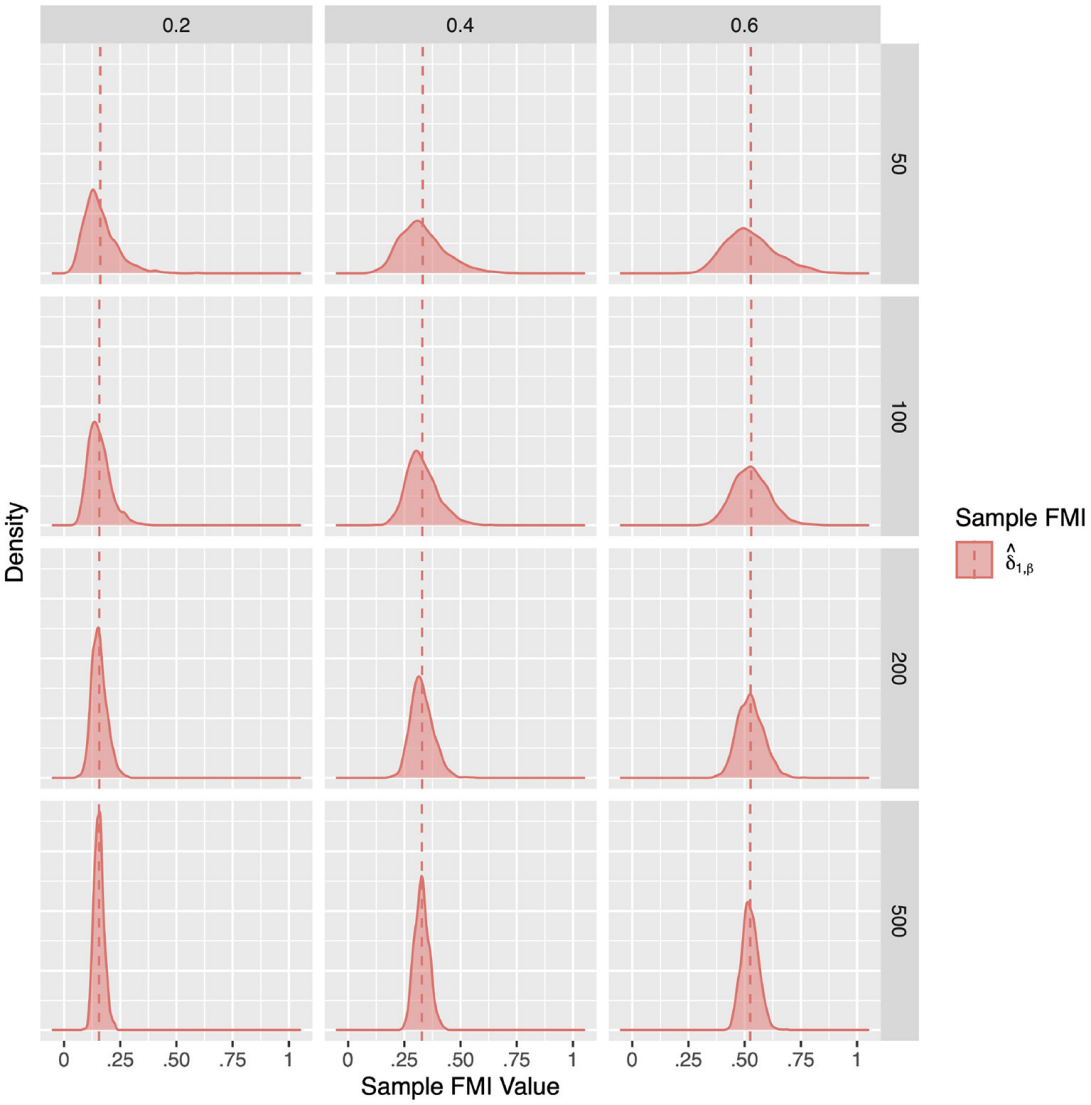
The sampling distribution of FMI for regression coefficients in MCAR. The panel rows correspond to sample sizes of *N* = 50, 100, 200, and 500. The panel columns correspond to per variable missing rates of 20, 40, and 60%, or overall population missing rates of 10, 20, and 30%. The population FMI value in each panel is given as a vertical black dotted line. The sampling distributions of the three estimates are virtually identical in simple regression.

**Figure 2:**
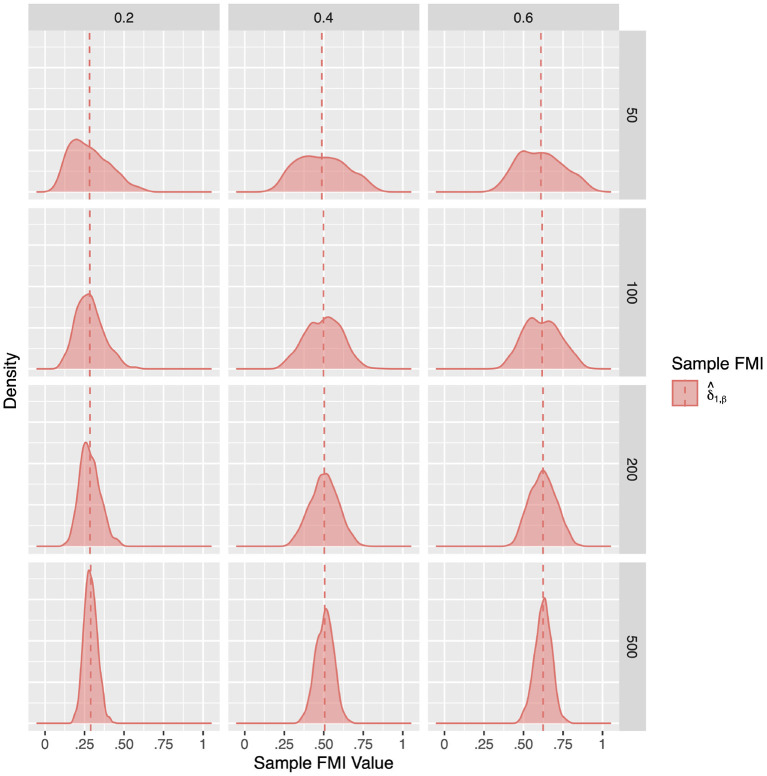
The sampling distribution of FMI for regression coefficients in linear MAR. The panel rows correspond to sample sizes of *N* = 50, 100, 200, and 500. The panel columns correspond to per variable missing rates of 20, 40, and 60, or overall population missing rates of 10, 20, and 30%. The population FMI value in each panel is given as a vertical black dotted line. The sampling distributions of the three estimates are virtually identical in simple regression.

**Figure 3:**
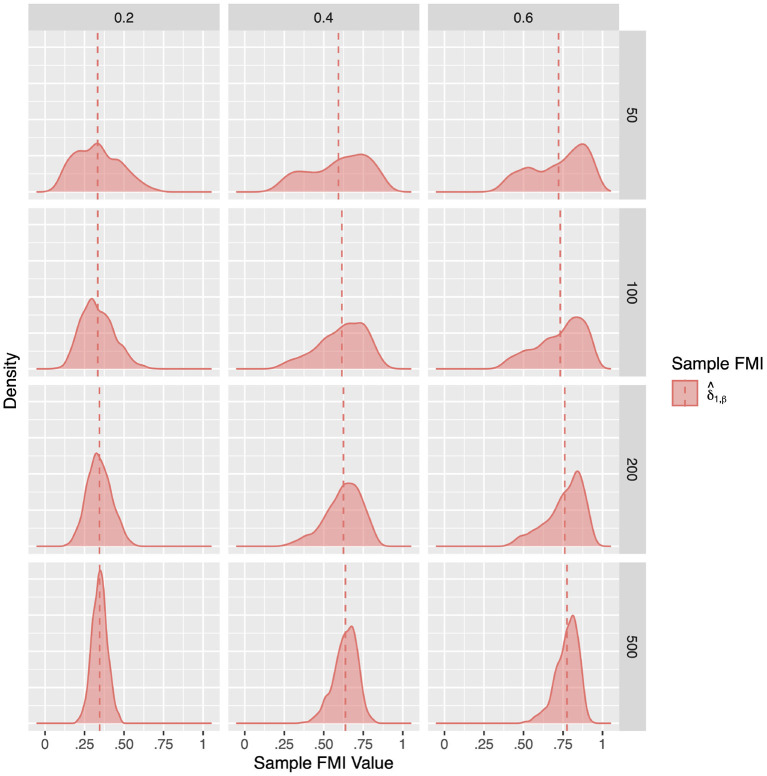
The sampling distribution of FMI for regression coefficients in nonlinear MAR. The panel rows correspond to sample sizes of *N* = 50, 100, 200, and 500. The panel columns correspond to per variable missing rates of 20, 40, and 60%, or overall population missing rates of 10, 20, and 30%. The population FMI value in each panel is given as a vertical black dotted line. The sampling distributions of the three estimates are virtually identical in simple regression.

For a more intuitive illustration of bias and variability, the smoothed densities of the 1,000 replications in each condition are shown in Figures 1–3. We can see that almost all sampling distributions centered around the population value, with the exception of the top right panels of Figure 3, which correspond to the small sample size, high missing rate, nonlinear MAR conditions. These distributions, however, do not pack very tightly around the population value, except when the sample size is 500, and only when either the missing rate was low, or the missing mechanism was MCAR.

#### 2.2.3. Model 2

In the two-factor model, not all replications were usable. In some cases, the model would fail to converge. In other cases, when FMIs were requested, *lavaan* would sometimes produce an error, leading to NAs or negative values for the FMI estimates. These issues were exacerbated by higher missing rates, and were particularly pronounced in ${\hat{\delta }}_{1,j}$ when the sample size was small. Under *N* = 100 and π_*mis*_=0.6 (overall missing rate 0.3), ${\hat{\delta }}_{1,j}$ would encounter more issues in more than 30% of the runs. In this regard, the best performing estimate was ${\hat{\delta }}_{3,j}$, which almost never produced negative values, and had the lowest rate of NA occurrences. However, even for ${\hat{\delta }}_{3,j}$, the rate of failed or improper estimates was often close to or above 10% at *N* = 100. At *N* = 200, ${\hat{\delta }}_{3,j}$ would encounter this issue at most 3% of the time, even when the per variable missing rate was 0.6. See Supplementary Tables 3, 4 for the rate of failed or improper estimates for the factor correlations and factor loadings, respectively. The following results were obtained by excluding all occurrences of NAs and negative values when aggregating the sample FMI estimates.

The sample FMIs of factor correlation (ϕ) were largely unbiased in MCAR, or when the mechanism was MAR but the sample size was 200 or above (see Tables 3–5). Bias was also generally small at *N* = 100 when the missing data mechanism was MCAR, but notable bias could occur in MAR-L and MAR-NL. Among the three estimates, ${\hat{\delta }}_{1,\phi }$ showed the largest amount of bias overall, severely overestimating the FMI (0.17 above the true value on average) when the missing data mechanism was nonlinear MAR and the per variable missing rate was π_*mis*_=0.6. ${\hat{\delta }}_{3,\phi }$ also showed some notable bias under *N* = 100, but only up to 0.11 in the worst case. ${\hat{\delta }}_{2,\phi }$ showed the least amount of bias, producing raw bias values very close to 0.05 or below across all the conditions.

**Table 3:** The bias, RMSE, and the 95% equal-tailed interval width of factor correlation FMIs under MCAR.

**π_*mis*_**	**N**	**Bias**			**RMSE**			**95% ETI width**		
		**${\hat{\delta }}_{1,\phi }$**	**${\hat{\delta }}_{2,\phi }$**	**${\hat{\delta }}_{3,\phi }$**	**${\hat{\delta }}_{1,\phi }$**	**${\hat{\delta }}_{2,\phi }$**	**${\hat{\delta }}_{3,\phi }$**	**${\hat{\delta }}_{1,\phi }$**	**${\hat{\delta }}_{2,\phi }$**	**${\hat{\delta }}_{3,\phi }$**
0.2	100	0.01	0.00	0.01	**0.10**	**0.06**	**0.06**	**0.31**	**0.23**	**0.24**
0.4	100	0.02	0.00	0.03	**0.16**	**0.12**	**0.11**	**0.67**	**0.44**	**0.39**
0.6	100	**0.07**	−0.02	0.05	**0.25**	**0.17**	**0.16**	**0.93**	**0.66**	**0.58**
0.2	200	0.00	0.00	0.00	0.04	0.04	0.04	0.17	0.16	0.15
0.4	200	−0.01	0.00	0.01	**0.09**	**0.08**	**0.08**	**0.34**	**0.30**	**0.29**
0.6	200	−0.02	−0.01	0.02	**0.15**	**0.12**	**0.12**	**0.56**	**0.46**	**0.43**
0.2	500	0.00	0.00	0.00	0.03	0.03	0.03	0.10	0.10	0.10
0.4	500	0.00	0.00	0.00	0.05	0.05	0.05	0.20	0.19	0.18
0.6	500	−0.01	−0.01	0.01	**0.08**	**0.07**	**0.07**	**0.30**	**0.28**	**0.28**
0.2	1000	0.00	0.00	0.00	0.02	0.02	0.02	0.07	0.07	0.07
0.4	1000	0.00	0.00	0.00	0.03	0.03	0.03	0.13	0.13	0.13
0.6	1000	−0.01	0.00	0.01	**0.05**	0.05	**0.05**	0.19	0.19	0.20

*π_mis_ is the probability of missing value in each variable with missingness. The overall missing rate is π_mis_/2. Distribution width is the distance between the 2.5 and 97.5 percentile in the sampling distributions. Bias values with magnitudes >0.05, RMSEs >0.05, and ETI widths >0.20 are indicated with bold fonts. The conditional formatting was applied prior to rounding*.

**Table 4:** The bias, RMSE, and the 95% equal-tailed interval width of factor correlation FMIs under linear MAR.

**π_*mis*_**	**N**	**Bias**			**RMSE**			**95% ETI width**		
		**${\hat{\delta }}_{1,\phi }$**	**${\hat{\delta }}_{2,\phi }$**	**${\hat{\delta }}_{3,\phi }$**	**${\hat{\delta }}_{1,\phi }$**	**${\hat{\delta }}_{2,\phi }$**	**${\hat{\delta }}_{3,\phi }$**	**${\hat{\delta }}_{1,\phi }$**	**${\hat{\delta }}_{2,\phi }$**	**${\hat{\delta }}_{3,\phi }$**
0.2	100	0.03	0.01	0.02	**0.12**	**0.07**	**0.07**	**0.38**	**0.26**	**0.25**
0.4	100	**0.07**	0.02	0.04	**0.20**	**0.13**	**0.13**	**0.87**	**0.49**	**0.45**
0.6	100	**0.12**	0.01	**0.08**	**0.28**	**0.19**	**0.17**	**0.91**	**0.73**	**0.61**
0.2	200	0.01	0.00	0.00	**0.06**	0.05	0.05	**0.20**	0.19	0.18
0.4	200	0.01	0.01	0.01	**0.10**	**0.09**	**0.08**	**0.38**	**0.33**	**0.31**
0.6	200	−0.01	0.00	0.04	**0.16**	**0.13**	**0.12**	**0.61**	**0.52**	**0.46**
0.2	500	0.00	0.00	0.00	0.03	0.03	0.03	0.12	0.11	0.11
0.4	500	0.00	0.00	0.00	**0.06**	**0.05**	**0.05**	**0.21**	**0.21**	0.20
0.6	500	0.00	0.00	0.01	**0.08**	**0.08**	**0.08**	**0.32**	**0.29**	**0.30**
0.2	1000	0.00	0.00	0.00	0.02	0.02	0.02	0.08	0.08	0.07
0.4	1000	0.00	0.00	0.00	0.04	0.04	0.04	0.14	0.14	0.14
0.6	1000	0.00	0.00	0.01	**0.05**	**0.05**	**0.05**	0.20	0.20	**0.20**

*π_mis_ is the probability of missing value in each variable with missingness. The overall missing rate is π_mis_/2. Distribution width is the distance between the 2.5 and 97.5 percentile in the sampling distributions. Bias values with magnitudes >0.05, RMSEs >0.05, and ETI widths >.20 are indicated with bold fonts. The conditional formatting was applied prior to rounding*.

**Table 5:** The bias, RMSE, and the 95% equal-tailed interval width of factor correlation FMIs under nonlinear MAR.

**π_*mis*_**	**N**	**Bias**			**RMSE**			**95% ETI width**		
		**${\hat{\delta }}_{1,\phi }$**	**${\hat{\delta }}_{2,\phi }$**	**${\hat{\delta }}_{3,\phi }$**	**${\hat{\delta }}_{1,\phi }$**	**${\hat{\delta }}_{2,\phi }$**	**${\hat{\delta }}_{3,\phi }$**	**${\hat{\delta }}_{1,\phi }$**	**${\hat{\delta }}_{2,\phi }$**	**${\hat{\delta }}_{3,\phi }$**
0.2	100	0.04	0.02	0.02	**0.12**	**0.07**	**0.08**	**0.42**	**0.28**	**0.26**
0.4	100	**0.10**	0.04	**0.06**	**0.23**	**0.15**	**0.14**	**0.90**	**0.54**	**0.47**
0.6	100	**0.17**	**0.05**	**0.11**	**0.30**	**0.20**	**0.19**	**0.90**	**0.76**	**0.59**
0.2	200	0.01	0.00	0.00	**0.06**	0.05	0.04	**0.22**	0.19	0.18
0.4	200	0.02	0.01	0.03	**0.13**	**0.11**	**0.09**	**0.48**	**0.40**	**0.34**
0.6	200	0.04	0.02	**0.06**	**0.19**	**0.15**	**0.14**	**0.82**	**0.56**	**0.47**
0.2	500	0.00	0.00	0.00	0.03	0.03	0.03	0.12	0.12	0.11
0.4	500	0.01	0.01	0.01	**0.07**	**0.06**	**0.05**	**0.28**	**0.26**	**0.20**
0.6	500	0.00	0.00	0.02	**0.11**	**0.10**	**0.08**	**0.43**	**0.37**	**0.32**
0.2	1000	0.00	0.00	0.00	0.02	0.02	0.02	0.08	0.08	0.07
0.4	1000	0.00	0.00	0.01	0.05	0.05	0.04	0.18	0.18	0.15
0.6	1000	0.00	0.00	0.01	**0.07**	**0.07**	**0.06**	**0.28**	**0.26**	**0.21**

*π_mis_ is the probability of missing value in each variable with missingness. The overall missing rate is π_mis_/2. Distribution width is the distance between the 2.5 and 97.5 percentile in the sampling distributions. Bias values with magnitudes >0.05, RMSEs >0.05, and ETI widths >0.20 are indicated with bold fonts. The conditional formatting was applied prior to rounding*.

**Figure 4:**
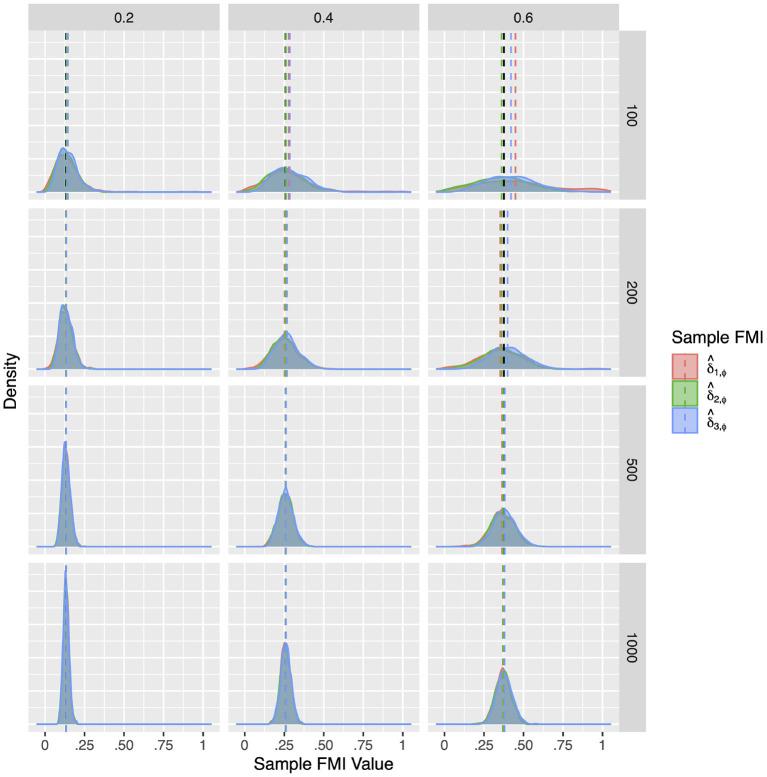
The sampling distribution of FMI for factor correlations in MCAR. The panel rows correspond to sample sizes of *N* = 50, 100, 200, and 500. The panel columns correspond to per variable missing rates of 20, 40, and 60%, or overall population missing rates of 10, 20, and 30%. The population FMI value in each panel is given as a vertical black dotted line.

**Figure 5:**
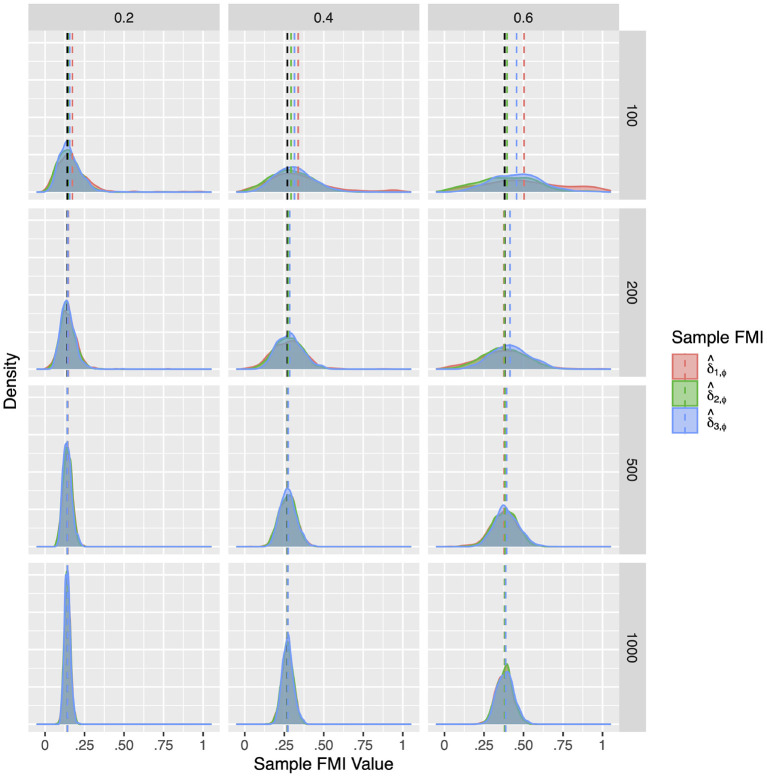
The sampling distribution of FMI for factor correlations in linear MAR. The panel rows correspond to sample sizes of *N* = 50, 100, 200, and 500. The panel columns correspond to per variable missing rates of 20, 40, and 60%, or overall population missing rates of 10, 20, and 30%. The population FMI value in each panel is given as a vertical black dotted line.

**Figure 6:**
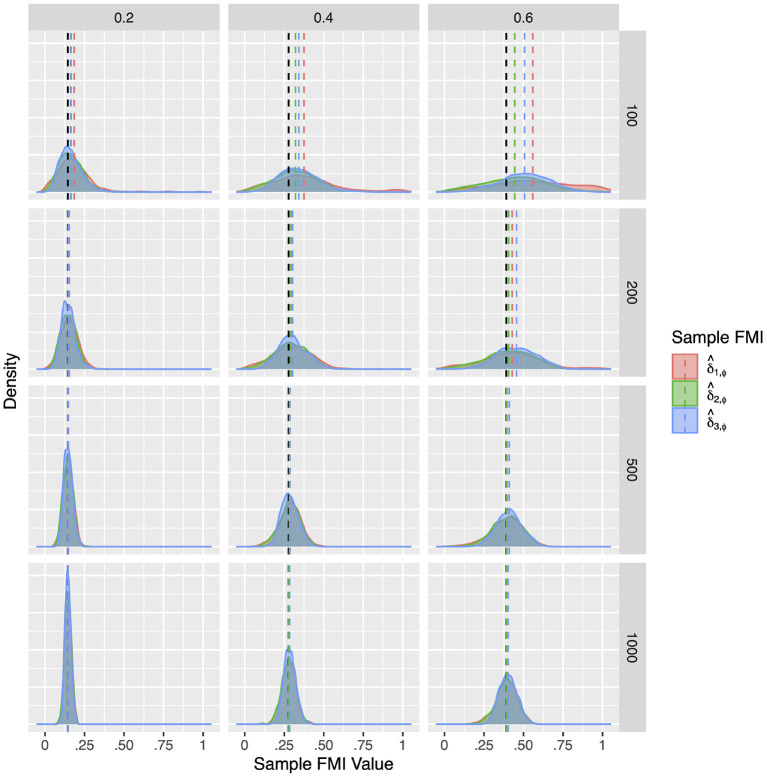
The sampling distribution of FMI for factor correlations in nonlinear MAR. The panel rows correspond to sample sizes of *N* = 50, 100, 200, and 500. The panel columns correspond to per variable missing rates of 20, 40, and 60%, or overall population missing rates of 10, 20, and 30%. The population FMI value in each panel is given as a vertical black dotted line.

Although the population FMIs of the factor correlations were quite low, they proved difficult to estimate precisely. As illustrated in Figures 4–6, the sampling distributions of the FMI estimates were very wide, and showed considerable bias at small sample sizes. Although the bias was absent at larger sample sizes, the estimates would only fall closely around the population value when the missing rate was low. As seen in Tables 3–5, when the per variable missing rate was π_*mis*_=0.4, a sample size of 500 is required for ${\hat{\delta }}_{3,\phi }$ to provide a precise estimate, with ${\hat{\delta }}_{1,\phi }$ and ${\hat{\delta }}_{2,\phi }$ giving worse performances. Under the high missing rate of π_*mis*_ = 0.6, a sample size of 1,000 was required. Overall, ${\hat{\delta }}_{3,\phi }$ gave the best performance, showing the lowest RMSE and 95% ETI width.

**Figure 7:**
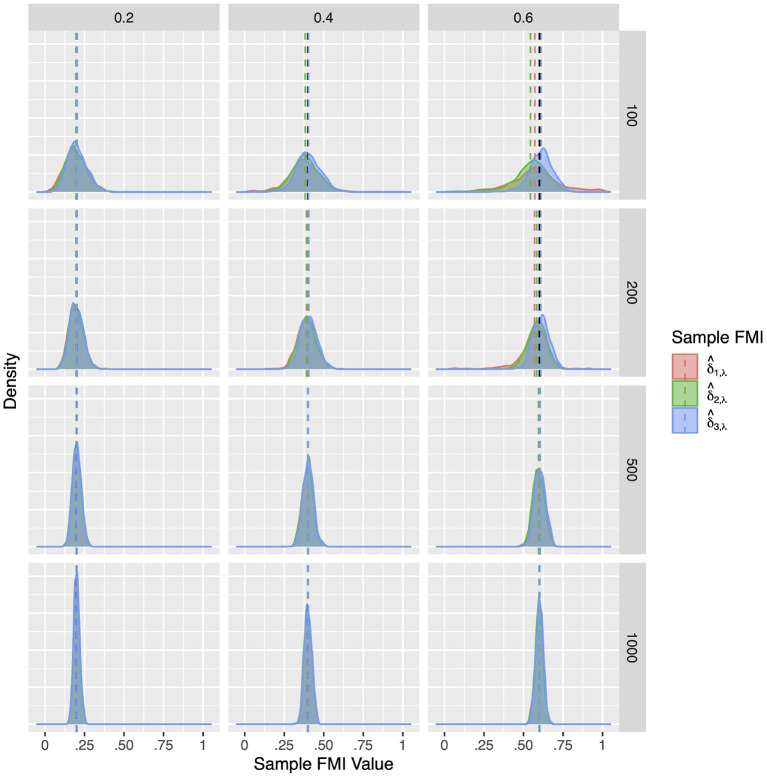
The sampling distribution of FMI for factor loadings in MCAR. The panel rows correspond to sample sizes of *N* = 50, 100, 200, and 500. The panel columns correspond to per variable missing rates of 20, 40, and 60%, or overall population missing rates of 10, 20, and 30%. The population FMI value in each panel is given as a vertical black dotted line.

**Figure 8:**
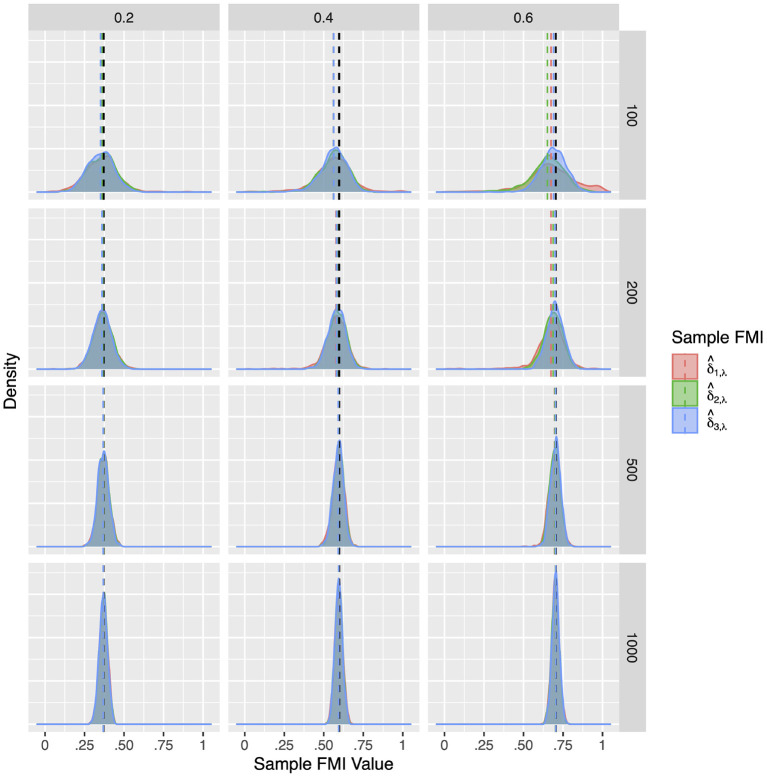
The sampling distribution of FMI for factor loadings in linear MAR. The panel rows correspond to sample sizes of *N* = 50, 100, 200, and 500. The panel columns correspond to per variable missing rates of 20, 40, and 60%, or overall population missing rates of 10, 20, and 30%. The population FMI value in each panel is given as a vertical black dotted line.

**Figure 9:**
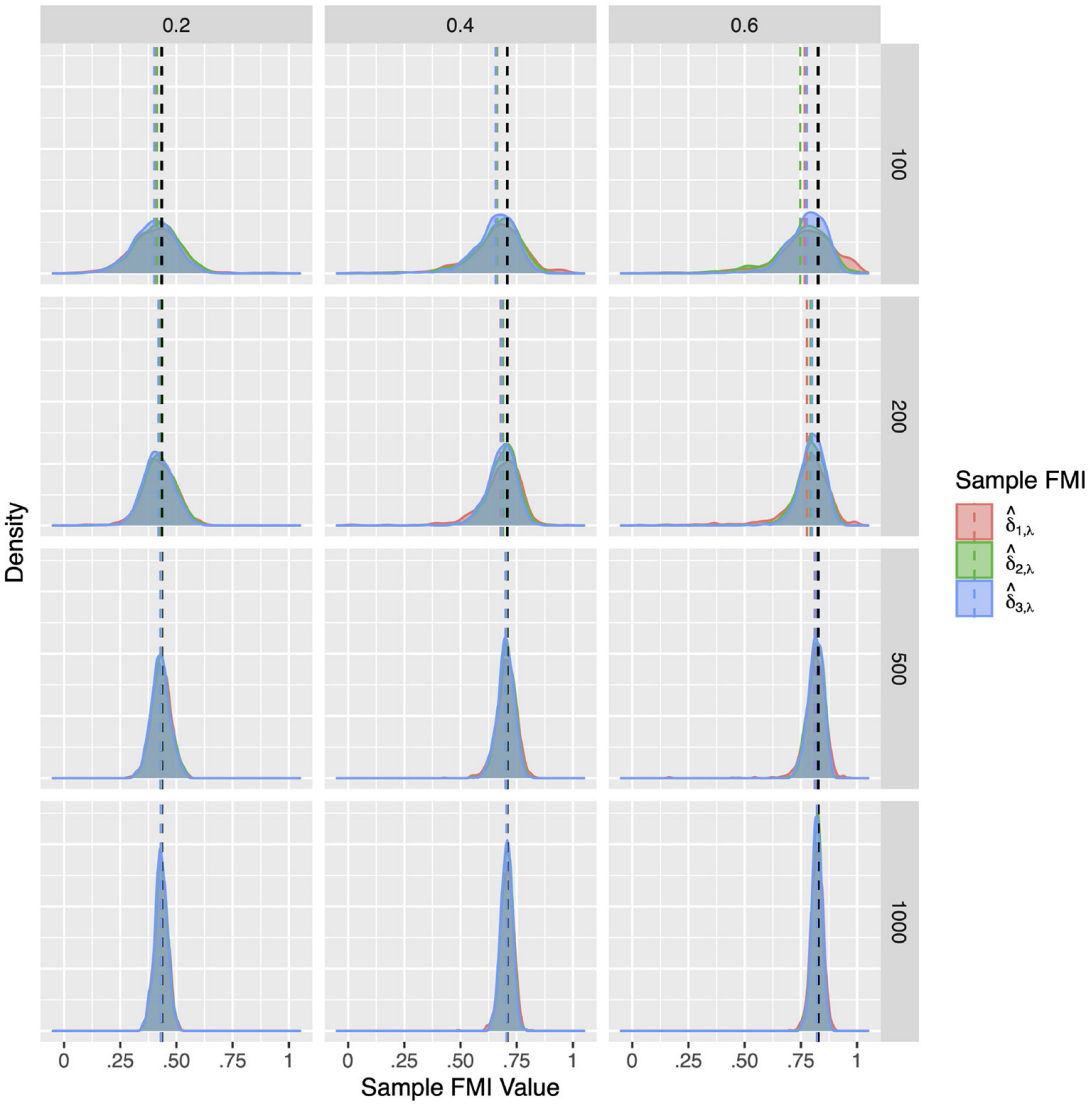
The sampling distribution of FMI for factor loadings in nonlinear MAR. The panel rows correspond to sample sizes of *N* = 50, 100, 200, and 500. The panel columns correspond to per variable missing rates of 20, 40, and 60%, or overall population missing rates of 10, 20, and 30%. The population FMI value in each panel is given as a vertical black dotted line.

Compared to the factor correlation FMIs, the factor loading FMIs performed much better in Model 2. Similar to the population simulation, here we report on the FMIs of the *X*_2_ loading on *F*1. The results for the other loadings *X*_4_, *Y*_2_, and *Y*_4_ are largely identical. Overall, as illustrated in Figures 7–9, the sample FMIs of factor loadings showed little bias and would typically fall much closer to the population values. As shown in Supplementary Tables 5–7, the bias, RMSE, and 95% ETI width were satisfactory for all three estimates when the sample size was *N* = 500 or greater. At *N* = 200, the FMIs only performed well when the missing mechanism was MCAR and the per variable missing rate was 0.2. At *N* = 100, all three estimates performed poorly, but ${\hat{\delta }}_{3,\lambda }$ was closer to acceptable performance, producing 95% ETI widths of 0.30 when ${\hat{\delta }}_{2,\lambda }$ would produce widths close to 0.50, and ${\hat{\delta }}_{1,\lambda }$ would produce widths close to 0.70. Once again, ${\hat{\delta }}_{3,\lambda }$ showed the best performance overall, with the lowest RMSE and 95% ETI width, and a similar bias to ${\hat{\delta }}_{2,\lambda }$.

## 3. Example Analysis

Here we provide an example on how to obtain FMIs from the *lavaan* package (version 0.6-7 and up) in *R*, using the Holzinger and Swineford (1939) dataset and simulated MCAR missing data. The example is adapted from Savalei and Rosseel (ress). The dataset, available through the *lavaan* package, contains cognitive performance test scores from 301 school children. The data for this example can be loaded into the *R* workspace using the following code.


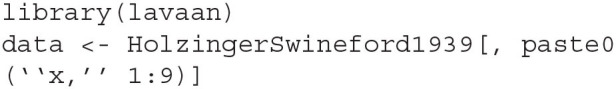


For the purpose of this demonstration, we will conduct a confirmatory factor analysis with three correlated factors: visual skills, verbal skills, and mental speed. Each factor is measured by three tests, with variable names x1 to x9 in the datasets, following a model given by the *lavaan* model syntax below.


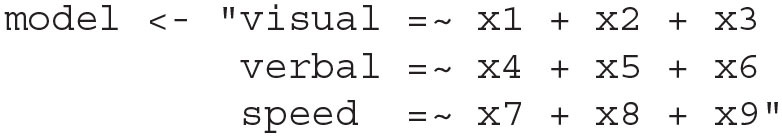


As the dataset does not contain missing data, we will introduce MCAR missingness by randomly removing 61 values from each variable independently, resulting in an overall missing rate of 20%.


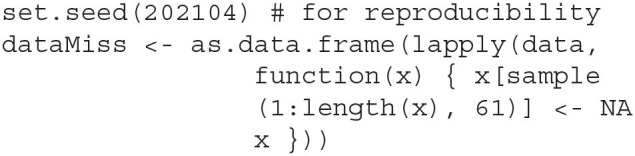


With the example data and model syntax prepared, we fit the data to the CFA model and obtain the FMIs with the following code.


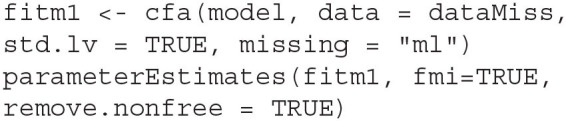


The option std.lv = TRUE fixes all variances of the latent factors to 1, allowing all loadings to be freely estimated, while the missing = ~ml~ asks *lavaan* to handle the missing data using FIML. The function parameterEstimates extracts the results from the model fit, where the option fmi = TRUE requests FMI estimates from *lavaan* alongside the parameter estimates. The remove.nonfree = TRUE option omits parameters that are not freely estimated from the output—In this case, the latent factor variances are not printed in the output table, as they were fixed to 1. By default, *lavaan* uses Hessian numeric estimation of the observed information matrix, which yields ${\hat{\delta }}_{1}$ for the FMI estimates. To produce ${\hat{\delta }}_{2}$, the observed.information input must be specified to request the analytic approximation of the information.


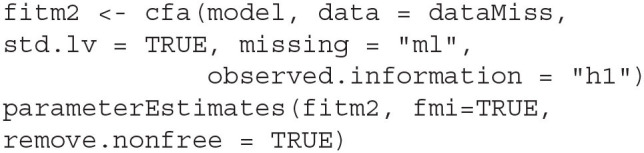


By default, the analytic approximation is based on structured information, to obtain ${\hat{\delta }}_{3}$, the h1.information input must be provided to request the unstructured information.


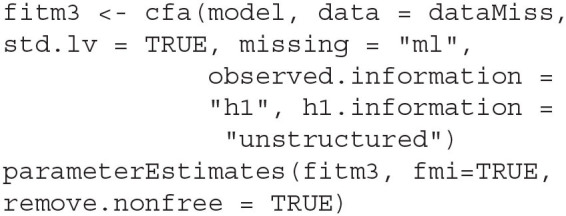


The three FMI estimates provide largely similar values (see Table 6). They agree on which loading estimates have the highest FMIs, namely the loadings of *X*_1_ (${\hat{\delta }}_{1}=0.29$, ${\hat{\delta }}_{2}=0.27$, ${\hat{\delta }}_{3}=0.29$), *X*_2_ (${\hat{\delta }}_{1}=0.26$, ${\hat{\delta }}_{2}=0.25$, ${\hat{\delta }}_{3}=0.28$), *X*_3_ (${\hat{\delta }}_{1}=0.28$, ${\hat{\delta }}_{2}=0.28$, ${\hat{\delta }}_{3}=0.31$), and *X*_7_ (${\hat{\delta }}_{1}=0.27$, ${\hat{\delta }}_{2}=0.27$, ${\hat{\delta }}_{3}=0.28$). The three estimates also agree on which factor correlation has the highest FMI, namely the correlation between visual skill and mental speed, ${\hat{\delta }}_{1}=0.23$, ${\hat{\delta }}_{2}=0.24$, ${\hat{\delta }}_{3}=0.29$. The FMI of the factor correlation between visual skill and mental speed also shows the largest difference among the FMI estimates, between 0.23 and 0.29. The three estimates disagree on which factor correlation has the lowest FMI, but the differences are small: For the correlation between visual and verbal skills, ${\hat{\delta }}_{1}=0.18$, ${\hat{\delta }}_{2}=0.15$, ${\hat{\delta }}_{3}=0.18$; for the correlation between verbal skill and mental speed, ${\hat{\delta }}_{1}=0.18$, ${\hat{\delta }}_{2}=0.18$, ${\hat{\delta }}_{3}=0.20$.

**Table 6:** Results from the example analysis.

**Parameter**	**Variables**	**${\hat{\delta }}_{1}$**	**${\hat{\delta }}_{2}$**	**${\hat{\delta }}_{3}$**
Loading	Visual, X1	0.29	0.27	0.29
Loading	Visual, X2	0.26	0.25	0.28
Loading	Visual, X3	0.28	0.28	0.31
Loading	Verbal, X4	0.24	0.22	0.24
Loading	Verbal, X5	0.17	0.17	0.19
Loading	Verbal, X6	0.19	0.18	0.19
Loading	Speed, X7	0.27	0.27	0.28
Loading	Speed, X8	0.23	0.24	0.26
Loading	Speed, X9	0.19	0.22	0.22
Factor correlation	Visual, verbal	0.18	0.15	0.18
Factor correlation	Visual, speed	0.23	0.24	0.29
Factor correlation	Verbal, speed	0.18	0.18	0.20

Overall, the highest FMI estimate in the model is 0.31. The associated WIF is $1/\sqrt{1-0.31}=1.2$, which indicates an estimated 20% increase of the width of the confidence interval due to missing data. Reporting these FMIs alongside analyses of empirical data will provide readers with a better sense of how much the presence of missing data has affected the efficiency of the parameter estimates, especially when comparing across studies where FMIs may differ even under the same missing rates. The full *R* code of this example is provided in Appendix A, and a summary of the lavaan options of the three estimates is given in Supplementary Table 1.

## 4. Discussion

The current simulation study suggests that a relatively large sample size may be necessary for the estimation of the FMIs. Sample FMI estimates were largely unbiased, even in very small samples with *N* = 50, which are typical of regression analyses. However, at such a small sample size, the estimates were imprecise, varying greatly from sample to sample, especially when the missing rate was high. When the missing rates were reasonably low (π_*mis*_ = 0.2, 0.4; corresponding to 10–20% overall missing rate), sample sizes of several hundreds, which are typical in structural equation models, were able to produce reasonably efficient estimates. However, at an overall missing rate of 30%, it would require sample sizes exceeding 1,000 to produce precise estimates. It is worth noting that we used very strong selection mechanisms in the MAR-L and MAR-NL conditions to contrast the results from MCAR. In applied settings, the MAR selection mechanism would typically be weaker, which would lead to sample FMI estimate performances that are closer to better performances we saw in the MCAR conditions.

The three estimates are identical for saturated models, such as regression. However, the choice makes a difference when the model is not saturated. In the two-factor model, ${\hat{\delta }}_{1,j}$, the estimate via numeric Hessian, showed a distinct disadvantage as it was more likely break down when the sample size was small, or when the missing rate was high. In contrast, ${\hat{\delta }}_{3,j}$, the analytic estimate based on the unstructured model, was much less likely to break down in all cases, and was more precise than ${\hat{\delta }}_{2,j}$. Although ${\hat{\delta }}_{3,j}$ occasionally showed a slightly higher bias than ${\hat{\delta }}_{2,j}$, which was based on the structured model, its performance was overall more favorable in these simulations.

The simulation study investigated FMI in FIML, but the results should generalize to FMI computed from MI with a large number of imputations. In MI, the FMI is conceptually given by the ratio of the between-imputation variance over the sum of the within- and between- imputation variances. As the number of imputations approaches infinity, this ratio becomes equivalent to the ratio of variance increase due to missing data over variance in the observed data as estimated from FIML. For simulation studies, FMI can be more computationally expensive in MI, as the estimate is produced in the final pooling stage of the analysis, and often requires a large number of imputations (more than 100) to achieve an acceptable level of accuracy (Harel, 2007). For substantive research, the researcher may simply choose between FIML and MI as the estimation method of FMI based on the missing data technique they are already using to produce the estimates of the model parameters.

As far as we are aware, this study is the first to look at the properties of sample FMIs computed using FIML. As such, we focused on two relatively simple and commonly used models, with three missing data mechanisms selected to contrast the impact of the specific mechanism on the FMI values and to stress that these values are not the same as the rates of missing data. Future research may wish to expand on the study conditions, for example, by controlling for the number of missing patterns, examining how changing the values of parameters in the model (such as the regression coefficient) would change the properties of the FMI estimates. It would also be worthwhile to investigate the relative performance of the three FMI estimates under incorrect models. When the model is wrong, the Hessian-based estimate, ${\hat{\delta }}_{1,j}$, is theoretically superior, as it is the only consistent estimate. However, whether this theoretical advantage would translate into a practical advantage needs to be examined in simulation studies. It would also be helpful to develop bootstrap SE/CI for the sample FMIs, so that researchers would have a better sense of the precision of the FMI estimates in their particular sample.

While our focus was on evaluating the properties of sample FMI estimates in terms how well they served as estimates of the corresponding population FMIs, the properties of population FMIs themselves may be of interest, and is a topic we are exploring in other work. In this ongoing work, we are finding that information loss can occur in unintuitive and unpredictable ways, and patterns in population FMIs observed in one context do not always generalize to other context. For example, based on the population-level FMI values we obtained for the conditions in this study, one may be tempted to conclude that the population FMIs of factor correlations are, in general, insensitive to missing data mechanisms (e.g., the middle rows of Table 1). However, this was not always the case. In conditions not reported here, when the indicators of *F*_1_ were all completely observed, and the indicators of *F*_2_ contained missingness conditioned on the indicators of *F*_1_, the FMI of the factor correlation became more sensitive to the missing data mechanism. Although the population quantities estimated by the sample FMIs may exhibit different patterns, we do not expect the sample FMI estimates themselves to show drastically different properties in these alternative scenarios.

The FMI estimates via FIML are available in most recent releases of *lavaan* (version 0.6-7 and up), and example *R* code for how to retrieve them is given in Appendix A. We recommend empirical researchers to routinely examine and report FMIs for key parameters in substantive analysis. FMIs capture the complex interplay between numerous factors such as missing rates, missing data mechanisms, and model parameters, sometimes in unintuitive ways. They can provide critical additional insights into how the standard errors, confidence intervals, and hypothesis tests may have been impacted by the presence of missing data. For methodologists conducting simulation studies with missing data, the population FMIs in the different study design conditions should be computed and reported, in order to provide better context of the performance of missing data techniques being studied.

## Data Availability Statement

The datasets presented in this study can be found in online repositories. The names of the repository/repositories and accession number(s) can be found at: https://osf.io/xyzt8/.

## Author Contributions

VS developed the equations, statistical properties, and details of implementation for FMI estimates. LC designed and carried out the simulation study and wrote the initial draft of the manuscript. All authors contributed to the revisions of the manuscript.

## Conflict of Interest

The authors declare that the research was conducted in the absence of any commercial or financial relationships that could be construed as a potential conflict of interest.

## Publisher's Note

All claims expressed in this article are solely those of the authors and do not necessarily represent those of their affiliated organizations, or those of the publisher, the editors and the reviewers. Any product that may be evaluated in this article, or claim that may be made by its manufacturer, is not guaranteed or endorsed by the publisher.

## Footnotes

^1^As the sample size approaches infinity, all parameter estimates approach their true values, and therefore all standard errors approach 0. Multiplying the standard errors by $\sqrt{n}$ makes them converge to nonzero numbers.

^2^See Appendix A for how to request FMI estimates in *lavaan*. We would like to thank Yves Rossel, the developer of *lavaan*, for implementing these variations in the computation of FMI, permitting us to carry out this study.
