# Supplementary material for: Three Sample Estimates of Fraction of Missing Information From Full Information Maximum Likelihood
Source: Front Psychol. 2021 Aug 26;12:667802. doi: 10.3389/fpsyg.2021.667802 (PMC8426626; doi:10.3389/fpsyg.2021.667802)
Supplement: Supplementary file 1 [file Data_Sheet_1.PDF]

Supplementary material for three sample estimates of fraction of missing information from full information maximum likelihood

Lihan Chen[1] and Victoria Savalei[1]

[1]University of British Columbia

Supplementary material for three sample estimates of fraction of missing information from full information maximum likelihood

This file contains the supplementary tables.

Table S1

Three options for FMI sample estimates in lavaan.

	Description	observed.information	h1.information
$\hat{\delta}_{1,j}$	Numeric Hessian	hessian (default)	(always structured)
$\hat{\delta}_{2,j}$	Structured Analytic	h1	structured (default)
$\hat{\delta}_{3,j}$	Unstructured Analytic	h1	unstructured

Note. See **?** and the *lavaan* documentation (**?**) for further details.

Table S2

Two-factor model parameters

	X_1	X_2	X_3	X_4	Y_1	Y_2	Y_3	Y_4
F1 loadings (λ)	.49	.49	.49	.49	0	0	0	0
F2 loadings (λ)	0	0	0	0	.49	.49	.49	.49
contained missing	no	yes	no	yes	no	yes	no	yes
conditioned by	none	$X_1 + X_3$	none	$X_1 + X_3$	none	$Y_1 + Y_3$	none	$Y_1 + Y_3$

Note. $F1$ and $F2$ are correlated at $\phi = .4$.

Table S3

The rate of failed or improper estimates in the sample FMIs of the factor correlation.

Mechanism	π_{mis}	N	Produced NAs			Negative Values		
			$\hat{\delta}_{1,j}$	$\hat{\delta}_{2,j}$	$\hat{\delta}_{3,j}$	$\hat{\delta}_{1,j}$	$\hat{\delta}_{2,j}$	$\hat{\delta}_{3,j}$
MCAR	0.2	100	13	10	10	47	5	0
MCAR	0.4	100	40	24	24	100	18	1
MCAR	0.6	100	210	97	83	145	82	3
MAR-L	0.2	100	29	24	24	61	9	1
MAR-L	0.4	100	106	80	80	93	12	1
MAR-L	0.6	100	206	128	109	124	65	5
MAR-NL	0.2	100	30	19	19	71	15	0
MAR-NL	0.4	100	138	85	84	96	52	2
MAR-NL	0.6	100	286	173	139	89	76	3
MCAR	0.2	200	0	0	0	8	0	0
MCAR	0.4	200	1	0	0	12	0	0
MCAR	0.6	200	40	2	2	77	13	0
MAR-L	0.2	200	1	1	1	5	1	0
MAR-L	0.4	200	1	2	2	25	2	0
MAR-L	0.6	200	56	13	12	78	14	1
MAR-NL	0.2	200	2	1	1	7	0	0
MAR-NL	0.4	200	12	2	2	45	7	0
MAR-NL	0.6	200	74	26	25	96	30	1

Note. Numbers are based on 1,000 replications. π_{mis} is the probability of missing value in each variable with missingness. The overall missing rate is $\pi_{mis}/2$. In conditions with $N = 500$ and $N = 1000$, the rates of failed or improper estimates are all below 1%.

Table S4

The rate of failed or improper estimates in the sample FMIs of the factor loadings.

Mechanism	π_{mis}	N	Produced NAs			Negative Values		
			$\hat{\delta}_{1,j}$	$\hat{\delta}_{2,j}$	$\hat{\delta}_{3,j}$	$\hat{\delta}_{1,j}$	$\hat{\delta}_{2,j}$	$\hat{\delta}_{3,j}$
MCAR	0.2	100	10	10	10	17	0	0
MCAR	0.4	100	31	24	24	34	1	0
MCAR	0.6	100	154	96	82	59	16	0
MAR-L	0.2	100	24	24	24	19	0	1
MAR-L	0.4	100	88	80	80	20	1	0
MAR-L	0.6	100	187	119	110	30	9	1
MAR-NL	0.2	100	24	19	19	14	0	0
MAR-NL	0.4	100	124	85	84	13	1	1
MAR-NL	0.6	100	243	181	140	12	13	0
MCAR	0.2	200	0	0	0	1	0	0
MCAR	0.4	200	1	0	0	3	0	0
MCAR	0.6	200	25	2	2	14	0	0
MAR-L	0.2	200	1	1	1	0	0	0
MAR-L	0.4	200	1	2	2	4	0	0
MAR-L	0.6	200	37	13	12	11	0	0
MAR-NL	0.2	200	2	1	1	1	0	0
MAR-NL	0.4	200	7	2	2	10	0	0
MAR-NL	0.6	200	69	27	25	11	0	0

Note. Numbers are based on 1,000 replications. As the loadings corresponding to variable containing missingness encountered failed or improper estimates at similar rates, the numbers here are based on the loading of X_2. π_{mis} is the probability of missing value in each variable with missingness. The overall missing rate is $\pi_{mis}/2$. In conditions with $N = 500$ and $N = 1000$, the rates of failed or improper estimates are all below 1%.

Table S5

The bias, RMSE, and the 95% equal-tailed interval width of factor loading FMIs under MCAR.

π_{mis}	N	Bias			RMSE			95% ETI Width		
		$\hat{\delta}_{1,\lambda}$	$\hat{\delta}_{2,\lambda}$	$\hat{\delta}_{3,\lambda}$	$\hat{\delta}_{1,\lambda}$	$\hat{\delta}_{2,\lambda}$	$\hat{\delta}_{3,\lambda}$	$\hat{\delta}_{1,\lambda}$	$\hat{\delta}_{2,\lambda}$	$\hat{\delta}_{3,\lambda}$
0.2	100	0.00	0.00	0.00	**0.08**	**0.06**	**0.06**	**0.29**	**0.24**	**0.23**
0.4	100	-0.02	-0.02	0.00	**0.12**	**0.09**	**0.08**	**0.44**	**0.34**	**0.30**
0.6	100	-0.03	**-0.06**	0.01	**0.16**	**0.13**	**0.07**	**0.73**	**0.48**	**0.30**
0.2	200	0.00	0.00	0.00	0.05	0.04	0.04	0.18	0.17	0.17
0.4	200	-0.01	0.00	0.01	**0.06**	**0.05**	**0.05**	**0.23**	**0.21**	**0.21**
0.6	200	-0.03	-0.02	0.01	**0.10**	**0.06**	**0.05**	**0.38**	**0.23**	0.20
0.2	500	0.00	0.00	0.00	0.03	0.03	0.03	0.11	0.11	0.10
0.4	500	0.00	0.00	0.00	0.04	0.03	0.03	0.14	0.14	0.14
0.6	500	-0.01	-0.01	0.00	0.04	0.04	0.04	0.14	0.14	0.13
0.2	1000	0.00	0.00	0.00	0.02	0.02	0.02	0.08	0.08	0.08
0.4	1000	0.00	0.00	0.00	0.02	0.02	0.02	0.09	0.09	0.09
0.6	1000	0.00	0.00	0.00	0.02	0.02	0.02	0.10	0.10	0.09

Note. π_{mis} is the probability of missing value in each variable with missingness. The overall missing rate is $\pi_{mis}/2$. Distribution width is the distance between the 2.5 and 97.5 percentile in the sampling distributions. The four loadings corresponding to variables containing missingness produced similar FMIs, and the FMIs for the loading of X_2 is reported here. Bias values with magnitudes larger than .05 are indicated with bold fonts. RMSEs equal to .05 or less, as well as ETI widths equal to .20 or less, are indicated with bold fonts.

Table S6

The bias, RMSE, and the 95% equal-tailed interval width of factor loading FMIs under linear MAR.

π_{mis}	N	Bias			RMSE			95% ETI Width		
		$\hat{\delta}_{1,\lambda}$	$\hat{\delta}_{2,\lambda}$	$\hat{\delta}_{3,\lambda}$	$\hat{\delta}_{1,\lambda}$	$\hat{\delta}_{2,\lambda}$	$\hat{\delta}_{3,\lambda}$	$\hat{\delta}_{1,\lambda}$	$\hat{\delta}_{2,\lambda}$	$\hat{\delta}_{3,\lambda}$
0.2	100	-0.01	-0.01	-0.02	**0.11**	**0.09**	**0.08**	**0.42**	**0.34**	**0.31**
0.4	100	-0.03	-0.03	-0.03	**0.13**	**0.10**	**0.09**	**0.50**	**0.34**	**0.32**
0.6	100	-0.03	**-0.05**	-0.01	**0.16**	**0.12**	**0.08**	**0.64**	**0.48**	**0.30**
0.2	200	-0.01	-0.01	-0.01	**0.06**	**0.06**	**0.06**	**0.24**	**0.24**	**0.22**
0.4	200	-0.02	-0.01	-0.01	**0.08**	**0.06**	**0.06**	**0.28**	**0.22**	**0.21**
0.6	200	-0.03	-0.02	0.00	**0.10**	**0.06**	**0.05**	**0.36**	**0.23**	**0.21**
0.2	500	0.00	0.00	0.00	0.04	0.04	0.04	0.15	0.14	0.13
0.4	500	0.00	0.00	0.00	0.04	0.03	0.03	0.14	0.13	0.13
0.6	500	-0.01	0.00	0.00	0.04	0.03	0.03	0.14	0.13	0.12
0.2	1000	0.00	0.00	0.00	0.03	0.03	0.03	0.10	0.11	0.10
0.4	1000	0.00	0.00	0.00	0.03	0.02	0.02	0.10	0.09	0.09
0.6	1000	0.00	0.00	0.00	0.02	0.02	0.02	0.10	0.09	0.09

Note. π_{mis} is the probability of missing value in each variable with missingness. The overall missing rate is $\pi_{mis}/2$. Distribution width is the distance between the 2.5 and 97.5 percentile in the sampling distributions. The four loadings corresponding to variables containing missingness produced similar FMIs, and the FMIs for the loading of X_2 is reported here. Bias values with magnitudes larger than .05 are indicated with bold fonts. RMSEs equal to .05 or less, as well as ETI widths equal to .20 or less, are indicated with bold fonts.

Table S7

The bias, RMSE, and the 95% equal-tailed interval width of factor loading FMIs under nonlinear MAR.

π_{mis}	N	Bias			RMSE			95% ETI Width		
		$\hat{\delta}_{1,\lambda}$	$\hat{\delta}_{2,\lambda}$	$\hat{\delta}_{3,\lambda}$	$\hat{\delta}_{1,\lambda}$	$\hat{\delta}_{2,\lambda}$	$\hat{\delta}_{3,\lambda}$	$\hat{\delta}_{1,\lambda}$	$\hat{\delta}_{2,\lambda}$	$\hat{\delta}_{3,\lambda}$
0.2	100	-0.02	-0.02	-0.03	**0.11**	**0.10**	**0.10**	**0.42**	**0.38**	**0.36**
0.4	100	-0.05	-0.05	**-0.05**	**0.14**	**0.12**	**0.11**	**0.54**	**0.42**	**0.37**
0.6	100	**-0.06**	**-0.08**	**-0.05**	**0.15**	**0.15**	**0.10**	**0.58**	**0.51**	**0.32**
0.2	200	-0.01	-0.01	-0.02	**0.08**	**0.07**	**0.07**	**0.29**	**0.26**	**0.25**
0.4	200	-0.03	-0.02	-0.03	**0.10**	**0.07**	**0.07**	**0.35**	**0.25**	**0.25**
0.6	200	**-0.05**	-0.03	-0.03	**0.12**	**0.08**	**0.06**	**0.47**	**0.24**	**0.21**
0.2	500	0.00	0.00	-0.01	0.04	0.04	0.04	0.17	0.17	0.16
0.4	500	0.00	-0.01	-0.01	0.04	0.04	0.04	0.17	0.15	0.15
0.6	500	-0.02	-0.01	-0.01	**0.06**	0.04	0.04	0.17	0.13	0.14
0.2	1000	0.00	0.00	-0.01	0.03	0.03	0.03	0.11	0.12	0.11
0.4	1000	0.00	0.00	0.00	0.03	0.03	0.03	0.12	0.10	0.10
0.6	1000	-0.01	-0.01	-0.01	0.03	0.02	0.02	0.11	0.09	0.09

Note. π_{mis} is the probability of missing value in each variable with missingness. The overall missing rate is $\pi_{mis}/2$. Distribution width is the distance between the 2.5 and 97.5 percentile in the sampling distributions. The four loadings corresponding to variables containing missingness produced similar FMIs, and the FMIs for the loading of X_2 is reported here. Bias values with magnitudes larger than .05 are indicated with bold fonts. RMSEs equal to .05 or less, as well as ETI widths equal to .20 or less, are indicated with bold fonts. These were applied prior to rounding.
