# Supplementary material for: Three Sample Estimates of Fraction of Missing Information From Full Information Maximum Likelihood
Source: Front Psychol. 2021 Aug 26;12:667802. doi: 10.3389/fpsyg.2021.667802 (PMC8426626; doi:10.3389/fpsyg.2021.667802)
Supplement: Supplementary file 2 [file Data_Sheet_2.pdf]

Appendices for three sample estimates of fraction of missing information from full information maximum likelihood

Lihan Chen[1] and Victoria Savalei[1]

[1]University of British Columbia

Appendices for three sample estimates of fraction of missing information from full information maximum likelihood

Appendix A

Example code for producing FMIs in *lavaan*:

```
library(lavaan)
# retrieving example data
data <- HolzingerSwineford1939[, paste0("x", 1:9)]
# specifying model for the example data
model <- "visual =~ x1 + x2 + x3
          verbal  =~ x4 + x5 + x6
          speed   =~ x7 + x8 + x9"
set.seed(202104) # for reproducibility
# producing missingness in example data
dataMiss <- as.data.frame(lapply(
                data, function(x) {
                        x[ sample(1:length(x), 61) ] <- NA
                    x }))

####################################
# code to compute delta 1
####################################
fitm1 <- lavaan::sem(model, data = dataMiss, std.lv = TRUE, missing = "ml")
est1 <- lavaan::parameterEstimates(fitm1, fmi = TRUE, remove.nonfree = TRUE)

# print delta 1 for loadings
est1[est1$op=="=~",]
# print delta 1 for factor correlations
est1[est1$op=="~~" & est1$lhs!=est1$rhs,]
```

```
# print delta 1 for intercepts
est1[est1$op=="~1",]

####################################
# code to compute delta 2
####################################
fitm2 <- lavaan::sem(model, data = dataMiss, missing = "ml", std.lv = TRUE,
             observed.information = "h1")
est2 <- lavaan::parameterEstimates(fitm2, fmi = TRUE, remove.nonfree = TRUE)

# print delta 2 for loadings
est2[est2$op=="=~",]
# print delta 2 for factor correlations
est2[est2$op=="~~" & est2$lhs!=est2$rhs,]
# print delta 2 for intercepts
est2[est2$op=="~1",]

####################################
# code to compute delta 3
####################################
fitm3 <- lavaan::sem(model, data = dataMiss, missing = "ml", std.lv = TRUE,
        observed.information = "h1", h1.information = "unstructured")
est3 <- lavaan::parameterEstimates(fitm3, fmi = TRUE, remove.nonfree = TRUE)

# print delta 3 for loadings
est3[est3$op=="=~",]
# print delta 3 for factor correlations
est3[est3$op=="~~" & est3$lhs!=est3$rhs,]
# print delta 3 for intercepts
```

```
est3[est3$op=="~1",]
```

Appendix B

This appendix provides the technical details for the three FMI estimates. It is based on Savalei and Rosseel (in press). The incomplete data log-likelihood in Equation 1 is maximized twice: once under the saturated model and once under the structured model. The number of parameters in the saturated model is $p^* = .5p(p+1) + p$, where p is the number of variables; following the notation of Yuan and Bentler (2000), these parameters will be referred to as β. The most common saturated model is one where all means, variances, and covariances are freely estimated, so that $\beta = ((\text{vech}\Sigma)', \mu')'$, where "vech" is the operator that stacks nonrendundant elements of a matrix into a vector columnwise. The number of parameters in the structured model is $q < p^*$; these parameters will be referred to as θ.

Denote by $\tilde{\beta} = ((\text{vech}\tilde{\Sigma})', \tilde{\mu}')'$ the saturated FIML estimates, obtained by maximizing Equation 1 with respect to β; these saturated FIML estimates are also known as the "EM" means and "EM" covariance matrix (stacked in a vector). Denote by $\hat{\theta}_{FIML}$ the structured FIML estimates, obtained by maximizing Equation 1 with respect to θ. Based on these estimates, we can also computed structured estimates of means and covariances, $\hat{\beta} = ((\text{vech}\hat{\Sigma})', \hat{\mu}')'$, where $\hat{\mu} = \mu(\hat{\theta}_{FIML})$ and $\hat{\Sigma} = \Sigma(\hat{\theta}_{FIML})$, as per the specified structural equation model.

The observed information matrix under the saturated model is needed in order to define the observed information matrix under the structured model. This matrix is $J_\beta = -\frac{1}{n}\frac{\partial^2 lnL(\beta)}{\partial\beta\partial\beta'} = \frac{1}{J}\sum_{i=1}^{J}\frac{n_j}{n}J_{\beta,j}$, where $j = 1, \ldots, J$ enumerates the missing data patterns that are possible under a particular MAR mechanism, n_j is the number of observations with the jth pattern, and $J_{\beta,j}$ is the pattern-specific observed information estimate, which is a function of the pattern-specific sample means and covariance matrix as well as of the population parameters μ and Σ (see eq. 5 in Savalei and Rosseel (in press) for an explicit expression).

The general expression for the observed information matrix under the structured model is $J_\theta = \Delta' J_\beta \Delta - \left(d'_\beta \otimes I_q\right) H$, where Δ is the $p^* \times q$ matrix of the SEM model first derivatives, H is the matrix of the SEM model second derivatives, I_q is the identity

matrix of order q, and d_β is the vector of first derivatives of the log-likelihood with respect to the saturated model.

The first and second derivatives in the general expression for J_θ can be approximated using various methods, leading to three different definitions for the observed information matrix estimates:

$$\hat{J}_\theta = \hat{\Delta}'\hat{J}_\beta\hat{\Delta} - \left(\hat{d}'_\beta \otimes I_q\right)\hat{H}$$

$$\hat{J}_{\theta,\mathrm{h1}} = \hat{\Delta}'\hat{J}_\beta\hat{\Delta}$$

$$\tilde{J}_\theta = \hat{\Delta}'\tilde{J}_\beta\hat{\Delta} - \left(\tilde{d}'_\beta \otimes I_q\right)\hat{H} = \hat{\Delta}'\tilde{J}_\beta\hat{\Delta},$$

where $\hat{\Delta}$ and $\hat{H}$ are the SEM model derivatives evaluated at $\hat{\theta}_{FIML}$; $\tilde{d}_\beta$ and $\hat{d}_\beta$ are the first derivatives of the log-likelihood with respect to the saturated model, but evaluated either at $\tilde{\beta}$ (i.e., at $\tilde{\mu}$ and $\tilde{\Sigma}$) or at $\hat{\beta}$ (i.e., at $\hat{\mu}$ and $\hat{\Sigma}$); and similarly $\tilde{J}_\beta$ and $\hat{J}_\beta$ are the negative second derivatives of the log-likelihood with respect to the saturated model (i.e., the saturated model's information matrix), evaluated at $\tilde{\beta}$ or at $\hat{\beta}$. Most SEM software programs have the first derivatives of the SEM model, contained in Δ, worked out analytically. However, the second derivatives contained H are less analytically tractable and thus the estimate $\hat{J}_\theta$, which relies on them, is harder to obtain analytically. However, because it is also the second derivative of the log-likelihood, most SEM software including *lavaan* obtain this estimate numerically rather than following the equation above. For this reason, we will refer to the estimate $\hat{J}_\theta$ as the numeric Hessian. On the other hand, the two approximations $\hat{J}_{\theta,\mathrm{h1}}$ and $\tilde{J}_\theta$ are obtained analytically using equations above. These approximations essentially discard the second term in the expression for $\hat{J}_\theta$, which depends on the first derivatives of the log-likelihood under the saturated model; these derivatives approach zero when the model is correct and this term tends to be very small relative to the first term. Thus, the analytic approximations assume the model is correct.

FMIs are defined by Equation 5. For incomplete data information (i.e., the denominator of Equation 5), we have estimates $\hat{J}_{Y,\theta}$, $\hat{J}_{Y,\theta,\mathrm{h1}}$, and $\tilde{J}_{Y,\theta}$, defined above, with subscript “Y" (indicating a computation based on incomplete data) added for clarity.

To compute FMIs according to Equation 5, we need estimates of complete and incomplete data information matrices, but we only have incomplete data. Fortunately, estimates of the information matrix with complete data can be obtained by using complete data formulas in equations above, but evaluating them at FIML estimates. In particular, *lavaan* can take the general expression for the complete data fit function (which is just a transformation of the complete data log-likelihood), evaluate it at the FIML estimates $\hat{\beta}$ and obtain numeric second derivatives to yield the estimate $\hat{J}_{X,\theta}$, the numeric Hessian estimating the would-be variability of parameter estimates if data were complete. Similarly, *lavaan* can take the expression for the complete data observed information matrix $J_{X,\theta}$, which, through its components, is a function of $\bar{x}$, S, μ, and Σ, and evaluate it either at $\{\tilde{\mu}, \tilde{\Sigma}, \tilde{\mu}, \tilde{\Sigma}\}$, resulting in $\tilde{J}_{Y,\theta}$, or evaluate it at $\{\tilde{\mu}, \tilde{\Sigma}, \hat{\mu}, \hat{\Sigma}\}$, resulting in $\hat{J}_{X,\theta,\text{h1}}$.

Using the same type of information matrix estimates in the numerator and denominator of Equation 5 will yield three local FMI estimate types:

$$\hat{\delta}_{1,j} = 1 - \frac{\{\hat{J}^{-1}_{X,\theta}\}_{jj}}{\{\hat{J}^{-1}_{Y,\theta}\}_{jj}}$$

$$\hat{\delta}_{2,j} = 1 - \frac{\{\hat{J}^{-1}_{X,\theta,\text{h1}}\}_{jj}}{\{\hat{J}^{-1}_{Y,\theta,\text{h1}}\}_{jj}}$$

$$\hat{\delta}_{3,j} = 1 - \frac{\{\tilde{J}^{-1}_{X,\theta}\}_{jj}}{\{\tilde{J}^{-1}_{Y,\theta}\}_{jj}}$$

References

Savalei, V., & Rosseel, Y. (in press). Computational options for standard errors and test statistics with incomplete nonnormal data. *Structural Equation Modeling*. Retrieved from https://psyarxiv.com/wmuqj/

Yuan, K.-H., & Bentler, P. M. (2000). Three likelihood-based methods for mean and covariance structure analysis with nonnormal missing data. *Sociological Methodology*, *30*, 165–200.
